# Electrostatic Interactions Explain the Higher Binding Affinity of the CR3022 Antibody for SARS-CoV-2 than the 4A8 Antibody

**DOI:** 10.1021/acs.jpcb.1c03639

**Published:** 2021-07-06

**Authors:** Hung Nguyen, Pham Dang Lan, Daniel A. Nissley, Edward P. O’Brien, Mai Suan Li

**Affiliations:** † Institute of Physics, Polish Academy of Sciences, al. Lotnikow 32/46, 02-668 Warsaw, Poland; ‡ Life Science Lab, Institute for Computational Science and Technology, Quang Trung Software City, Tan Chanh Hiep Ward, District 12, Ho Chi Minh City, Vietnam; § Faculty of Physics and Engineering Physics, VNUHCM-University of Science, 227, Nguyen Van Cu Street, District 5, Ho Chi Minh City, Vietnam; ∥ Department of Statistics, University of Oxford, Oxford Protein Bioinformatics Group, Oxford OX1 2JD, United Kingdom; ⊥ Department of Chemistry, Penn State University, University Park, Pennsylvania 16802, United States; # Bioinformatics and Genomics Graduate Program, The Huck Institutes of the Life Sciences, Penn State University, University Park, Pennsylvania 16802, United States; ∇ Institute for Computational and Data Sciences, Penn State University, University Park, Pennsylvania 16802, United States

## Abstract

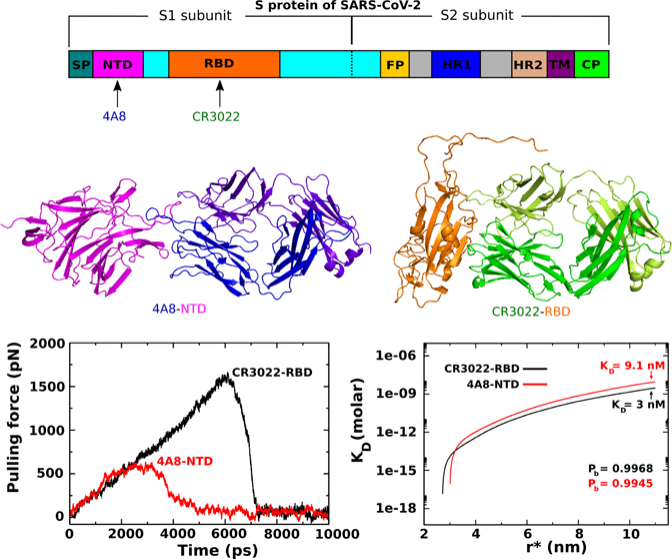

A structural understanding of the mechanism by which antibodies bind SARS-CoV-2 at the atomic level is highly desirable as it can tell the development of more effective antibodies to treat Covid-19. Here, we use steered molecular dynamics (SMD) and coarse-grained simulations to estimate the binding affinity of the monoclonal antibodies CR3022 and 4A8 to the SARS-CoV-2 receptor-binding domain (RBD) and SARS-CoV-2 N-terminal domain (NTD). Consistent with experiments, our SMD and coarse-grained simulations both indicate that CR3022 has a higher affinity for SARS-CoV-2 RBD than 4A8 for the NTD, and the coarse-grained simulations indicate the former binds three times stronger to its respective epitope. This finding shows that CR3022 is a candidate for Covid-19 therapy and is likely a better choice than 4A8. Energetic decomposition of the interaction energies between these two complexes reveals that electrostatic interactions explain the difference in the observed binding affinity between the two complexes. This result could lead to a new approach for developing anti-Covid-19 antibodies in which good candidates must contain charged amino acids in the area of contact with the virus.

## 1 Introduction

The first outbreak of coronavirus disease 2019 was known in Wuhan, China, in December 2019; then, it became a global pandemic in March 2020 and was named Covid-19.^1^ Covid-19 is caused by a novel coronavirus, a severe acute respiratory syndrome coronavirus 2 (SARS-CoV-2).^2^ As of 21 March 2021, Covid-19 has resulted in a total of more than 123 million infections and more than 2.7 million deaths (https://coronavirus.jhu.edu/map.html).

Drugs, vaccines, and antibodies can be used to combat Covid-19. However, no new medication has been developed at this time, although several older drugs have been reported to be effective. For example, FDA-approved remdesivir^3^ and dexamethasone^4^ improve the conditions of severe patients, but they may weaken the immune system.^5^ Currently, vaccines developed by various companies such as Pfizer-BioNTech, Moderna, and AstraZeneca are being widely used, but there are cases of resistance and their side effects have not been fully studied. More importantly, Johnson&Johnson (J&J) and Novavax vaccines may not be effective against the South Africa B.1.351 variant of SARS-CoV-2.^6^ Antibodies isolated from the plasma of recovered SARS-CoV-2 patients have been proven to effectively treat new patients.^7^ However, the amount of plasma available will be insufficient for the growing number of cases, which requires the production of antibodies on a larger scale.

**Figure 1:**
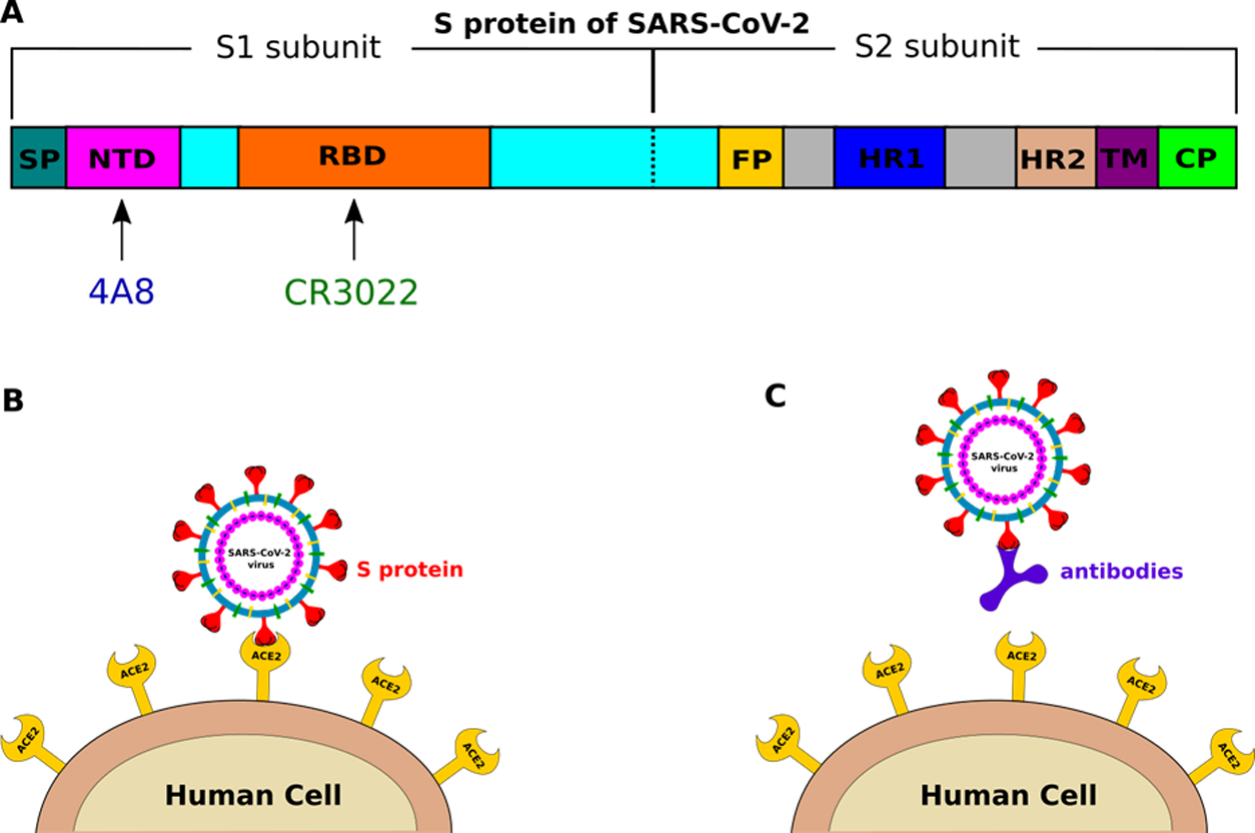
(A) Schematic description of the S protein of SARS-CoV-2, which consists of subunits S1 and S2. Monoclonal antibody (mAb) can bind to RBD, NTD, and FP (fusion peptide). (B) S protein of SARS-CoV-2 binds to human ACE2 before its entry to cells. (C) Antibody binds to the S protein, preventing the virus from entering cells.

Coronaviruses are spherical in shape with protruding molecules from the viral surface called spike (S) proteins (Figure 1A,B). The S protein decorates the surface of coronavirus and plays a pivotal role in viral replication by binding to human angiotensin-converting enzyme 2 (ACE2).^8^ Antibodies can bind with the S protein, preventing the virus from entering cells (Figure 1C). The S protein is cleaved into the N-terminal S1 subunit and C-terminal S2 subunit by host proteases and changes conformation from the prefusion to the postfusion state^9^^,^^10^ (Figure 1A). The S1 and S2 subunits comprise an extracellular domain and a single transmembrane helix that function to mediate receptor binding and membrane fusion, respectively.^9,11^ Importantly, S1 contains the N-terminal domain (NTD) and the receptor-binding domain (RBD), which are critical in determining tissue tropism and host range.^12^^,^^13^ The NTD may recognize specific sugar moieties upon initial attachment^14,15^ and plays an important role in the pre- to postfusion transition of the S protein.^16,17^ RBD binding to human cells is a critical step, allowing coronaviruses to enter cells to cause infection.^18,19^ Since most of the antibodies bind to either NTD or RBD (Figure 1A), understanding the interactions of antibodies with these regions of SARS-CoV-2 at the atomic level is important for Covid-19 therapies and vaccinations. There are many antibodies that target SARS-CoV-2,^7^ but in this study, we focus on two antibodies CR3022 and 4A8 because they hold promise for treating Covid-19 (see below); our computational results may offer insight into the controversial experimental results for these two antibodies, and they bind to different regions of the S protein, allowing more targets to be explored.

CR3022, a neutralizing antibody that targets RBD of old SARS-CoV, was previously isolated from a convalescent SARS patient.^20^ Recent studies indicated that CR3022 can also bind to RBD of SARS-CoV-2^21^ (Figure 1A), suggesting a potential opportunity to uncover a cross-reactive epitope. Yuan et al. showed that CR3022 can neutralize SARS-CoV but not SARS-CoV-2 RBD at a maximum concentration of 400 μg/mL.^21^ Namely, CR3022 binds to SARS-CoV RBD (*K*_D_ = 1 nM) with a much higher affinity than it does to SARS-CoV-2 RBD (*K*_D_ = 115 nM) (Table 1), implying that CR3022 could not be a candidate for the treatment of SARS-CoV-2.^21^ In contrast to Yuan et al., Tian and colleagues found that CR3022 binds efficiently with SARS-CoV-2 RBD (*K*_D_ = 6.3 nM)^22^ (Table 1), suggesting that CR3022 alone or in combination with other neutralizing antibodies has potential for prevention and treatment of Covid-19.

**Table 1:** *K*_D_ (nM) of CR3022–RBD and 4A8–NTD Complexes Obtained by Experiments and Simulations

complex	experiment results (*K*_D_)	our simulation results (*K*_D_)
4A8–NTD (PDB ID: 7C2L)	92.7 nM (Chi et al.)^23^	9.1 nM
CR3022–RBD (PDB ID: 6W41)	6.3 nM (Tian et al.)^22^	3.0 nM
	115.0 nM (Yuan et al.)^21^	

In contrast to CR3022, 4A8 is a monoclonal antibody that targets SARS-CoV-2 NTD (Figure 1A) and does not bind RBD.^23^ Chi et al. reported that 4A8 is a good candidate for the treatment of Covid-19, as it has a strong neutralizing capacity against both authentic and pseudotyped SARS-CoV-2 NTD (*K*_D_ = 92.7 nM)^23^ (Table 1). The epitope of 4A8 on SARS-CoV-2 S protein NTD was determined by cryoelectron microscopy, and its structure in complex with the S protein was obtained with an overall resolution of 3.1 Å and a local resolution of 3.3 Å at the 4A8–SARS-CoV-2 NTD interface.^23^ These findings indicate that 4A8 is also a promising therapeutic antibody against SARS-CoV-2 infection.

This work has two goals: (i) to understand the molecular mechanism of CR3022 and 4A8 binding to the S protein and their ability to treat Covid-19 and (ii) to shed light on the controversy between the two experimental groups.^21,22^ Using all-atom steered molecular dynamics and coarse-grained simulations, we showed that CR3022 binds strongly to SARS-CoV-2 RBD, especially compared to the binding affinity of 4A8 to SARS-CoV-2 NTD. Our results are consistent with the experimental data of Tian et al.^22^ and Chi et al.^23^ but not with the data of Yuan et al.^21^ (Table 1). The difference between the *K*_D_ values obtained by Tian et al.^22^ and Yuan et al.^21^ for CR3022 is likely due to the different experimental conditions they used (see below for more details). Therefore, our models perform better under the in vitro conditions adopted by Tian et al.^22^

One of our most important results is that electrostatic interactions are more dominant than van der Waals interactions in antibody binding to the S protein. This may lead to a new strategy for antibody design, according to which the binding region of potential therapeutic agents should be rich in charged residues.

## 2 Materials and Methods

### 2.1 Preparation of Input Protein Structures

**Figure 2:**
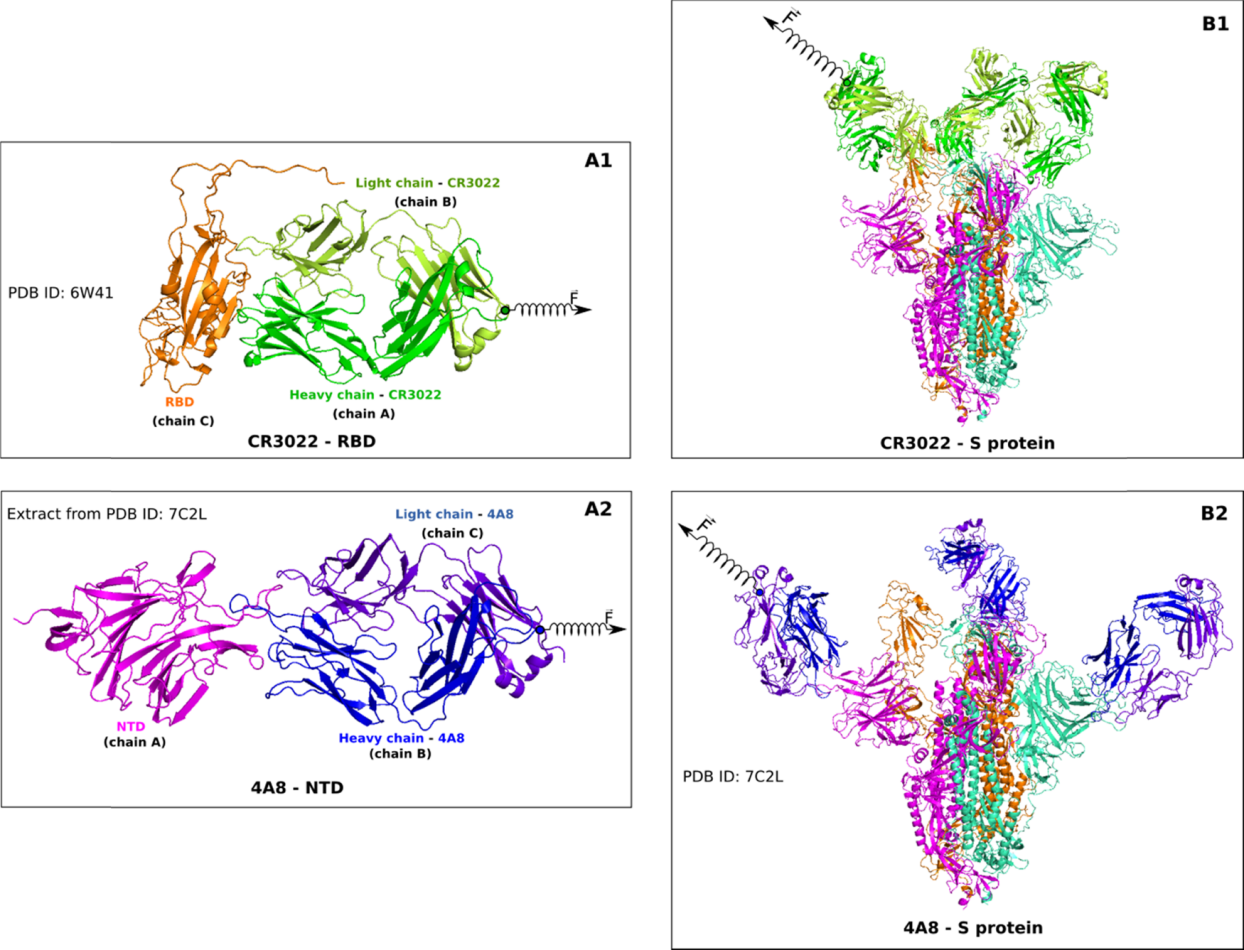
(A1) Structure of the CR3022–RBD complex, retrieved from PDB with ID 6W41. RBD is orange, while green and lemon describe CR3022. (A2) 4A8–NTD complex was extracted from the PDB structure with ID 7L2C. NTD is magenta, and blue and purple-blue refer to 4A8. (B1) Structure of the CR3022–S protein complex, which was obtained by docking CR3022 to the PDB structure 7L2C without 4A8. (B2) PDB structure of the 4A8–S protein complex (PDB ID: 7C2L). The pulling direction in SMD simulations is shown with a spring. The plot was made by the PyMOL 2.0 package.^43^

The structures of CR3022–SARS-CoV-2 RBD (CR3022–RBD) and 4A8–SARS-CoV-2 NTD (4A8–NTD) were extracted from the Protein Data Bank with PDB ID 6W41(21) (for CR3022–RBD) and 7L2C^23^ (for 4A8–NTD) (Figure 2A1, A2). The 4A8–S protein complex (4A8–S protein) was taken from PDB (ID: 7C2L, Figure 2B2), while the CR3022–S protein complex (CR3022–S protein) was constructed by the ClusPro server,^24,25^ where CR3022 was docked to SARS-CoV-2 RBD of the S protein in the open state (was taken from 7L2C without 4A8). The docking result of CR3022 binding to SARS-CoV-2 RBD was then made by a structural alignment with CR3022–RBD (ID: 6W41), and the root-mean-square deviation (RMSD) was 0.1 nm. The missing residues were added by the Modeler package.^26^ The structure of CR3022–S protein is shown in Figure 2B1.

### 2.2 All-Atom Molecular Dynamics Simulations

The simulation process of the complexes was performed by CHARMM36^27^ and AMBER99SB-DISP^28^ force fields implemented in the GROMACS 2016 package^29^ at 310 K and an isotropic pressure of 1 bar, which was obtained using the v-rescale and Parrinello–Rahman algorithms.^30,31^ The Tip3p water model^32^ was used in all simulation systems. Bond lengths were constrained by the linear constraint solver (LINCS) algorithm,^33^ which allows us to use a time step of 2 fs. The electrostatic and van der Waals interactions were used to depict nonbonded interactions, with the nonbonded interaction pair list being updated every 10 fs using a cutoff of 1.4 nm. The particle mesh Ewald algorithm^34^ was used to treat the long-range electrostatic interactions. Periodic boundary conditions were applied in all directions. From these structures, the energy of the system was minimized by the steepest-descent algorithm; then, a short 2 ns MD simulation was performed in the NVT ensemble, which was followed by 3 ns of NPT simulation. Next, a 100 ns production MD simulation was run with an integration time step of 2 fs and the leap-frog algorithm.^35^ The “gmx_mpi cluster” tool in the GROMACS 2016 package was used to collect a set of five trajectories for each system to perform steered molecular dynamics simulations.^36−39^

### 2.3 Steered Molecular Dynamics

To investigate CR3022 and 4A8 binding to the S protein, we used SMD, which is as useful as other computationally demanding MD methods in accessing relative binding affinities of ligands.^40−42^ This method was also helpful to analyze the interaction between SARS-CoV RBD and human ACE2.^39^

We carried out SMD simulations to pull CR3022 and 4A8 from their RBD and NTD binding regions using the CHARMM36 force field. For each complex, five different trajectories were run at pulling speeds of *v* = 0.5, 1.5, and 5 nm/ns. To check the robustness of the results to a change in the force field, additional simulations were conducted using the Amber99sb-disp force field.

Rectangular boxes with dimensions of 10 × 6 × 23 nm^3^ and 7 × 7 × 25 nm^3^ were used for CR3022–RBD and 4A8–NTD, respectively. However, for the much larger CR3022–S protein and 4A8–S protein complexes, boxes of 18.4 × 21.4 × 37 and 26 × 27.4 × 37 nm^3^ dimensions were used to allow enough room to pull CR3022 and 4A8 from the binding region. All complexes were immersed in a 0.15 M sodium chloride salt solution to neutralize the total charge.

A spring is attached to a dummy atom on one side and on the other side to the center of mass (CoM) of the antibody (Figure 2). The dummy atom is then pulled from its initial position along the line connecting the antibody CoM and CoM of RBD or NTD at a constant speed *v*. The complexes were rotated so that the unbinding pathway CR3022 and 4A8 is along the *z*-axis (Figure 2), which is displayed using the PyMOL 2.0 package.^43^ The pulling force is calculated according to the following equation
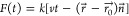
1where *k* is the stiffness of the spring connecting the dummy atom and the antibody CoM, *n⃗* is the normal direction of pulling, and *r⃗* and  are the positions of the system at time *t* and initial time, respectively. Spring constant *k* was set to 600 kJ/(mol nm^2^) (≈ 1020 pN/nm), which is a typical value used in atomic force microscopy (AFM) experiments.^44^

Using the force–displacement profile obtained from the SMD simulation, the pulling work (*W*) performed by the antibody was estimated using the trapezoidal rule
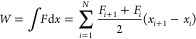
2where *N* is the number of simulation steps and *F_i_* and *x_i_* are the forces experienced by the target and position at step *i*.

To estimate the nonequilibrium binding free energy (Δ*G*), we used Jarzynski’s equality^45^ extended to the case when the external force grows at a constant speed *v*(46)
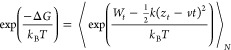
3where ⟨...⟩_*N*_ is the average over *N* trajectories, *z*_*t*_ is the time-dependent displacement, and *W_t_* is the nonequilibrium work at time *t*, i.e., *W_t_* = *W*(*t*), where *W* is defined by eq 2.

In general, using eq 3, we can extract the equilibrium free energy when the number of simulations is large enough. However, in this study, when the pulling is not slow enough and the number of SMD runs is limited, we can only evaluate the nonequilibrium binding and unbinding energy barriers separating the transition state (TS) from the bound state at *t*_0_ and the unbound state at *t*_end_, respectively.^40^

### 2.4 Definition of Hydrogen Bond and Nonbonded Contact

A hydrogen bond (HB) is formed when the distance between donor D and acceptor A is less than 0.35 nm, the H-A distance is less than 0.27 nm, and the D-H-A angle is larger than 135°. A nonbonded contact (NBC) between two residues of an antibody and a protein is formed when the shortest distance between their atoms is within 0.39 nm. The two-dimensional (2D) contact networks of HBs and NBCs of CR3022–RBD and 4A8–NTD were constructed using the LIGPLOT package.^47^

### 2.5 Coarse-Grained Simulations

#### 2.5.1 Coarse-Grained Model

The potential energy of the system in this model is given by the following expression:^48^
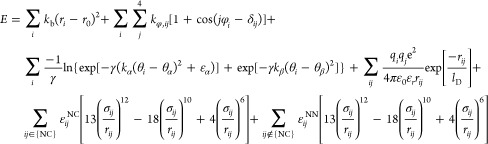
4where the terms in order represent the potential energy contributions from bonds, dihedrals, bond angles, electrostatics, native contacts, and non-native interactions, respectively.^49−51^ The functional forms of the first three terms have been described in detail previously.^49−51^

The Debye–Huckel theory was employed to model the electrostatic interactions with Debye screening length *l*_D_ = 1 nm and a dielectric constant of 78.5.^52^ Lysine and arginine residues were assigned a charge of +1e, glutamate and aspartate were assigned a charge of −1e, and all other residues were assigned a charge of zero. The contribution from attractive native interactions was computed using the 12-10-6 potential of Karanicolas and Brooks.^53^ Collision diameters σ_*ij*_ between the *C*_α_ interaction sites were set equal to their distance in the crystal structure divided by 2^1/6^. The value of ε_NC_, which sets the depth of the energy minimum for a native contact, was calculated to be ε_NC_ = *n_ij_* ε_HB_ + ηε*_ij_*. Here, ε_HB_, and ε*_ij_* represent energy contributions arising from hydrogen bonding and van der Waals between residues *i* and *j* from the all-atom structure of the protein, respectively. The number of hydrogen bonds *n_ij_* formed between residues *i* and *j* is defined using STRIDE software,^54^ and ε_HB_ is set equal to 0.75 kcal/mol. Intraprotein and interprotein Lennard–Jones (LJ) contacts are defined using a cutoff distance of 0.45 nm between any two heavy atoms of a pair of residues. The value of ε*_ij_* is based on the Betancourt–Thirumalai pairwise statistical potential.^55^ While the other terms are transferable among proteins, the LJ well depths for native contacts ε*_ij_* are scaled by a factor η to reproduce the stability of the modeled structures.^48^ η values for intraprotein and interprotein interactions of native contacts will be discussed below. All non-native interactions are treated by the final term in the summation using ε*_ij_* = 0.000132 kcal/mol and σ_*ij*_ values used as previously described.^56^

#### 2.5.2 Parameterizing the LJ Well Depths for Protein Stability

We applied a previously published training set and parameter tuning procedure to select realistic intra- and interprotein energy scales for native contacts.^56^ Sets of ten 1 μs simulations were run with values of η = 1.442, 1.759, 2.480 and 1.235, 1.507, 2.124 for domain and interface of antibodies, respectively, while 1.114, 1.359, 1.916 for SARS-CoV-2 RBD domain and 1.442, 1.759, 2.480 for SARS-CoV-2 NTD domain. The smallest η values were chosen that results in a model that is folded ≥98% of the time in each simulation, and a given conformation was considered to be folded when its fraction of native contacts is greater than 0.69. To assess how strong 4A8 binds to the SARS-CoV-2 NTD domain as compared to CR3022 to the SARS-CoV-2 RBD domain, we plan to employ the same set of parameters for two complexes. For this reason, we set η = 1.442 in simulations instead of 1.359 for the RBD domain, as listed in Table S2. The interaction energy scale for contacts between antibodies and SARS-CoV-2 domains was assigned by an η value of 1.4 to reproduce experimental *K*_D_ values on the order of nm.

#### 2.5.3 Replica Exchange Umbrella Sampling (REX-US) Simulations

Coarse-grained protein simulations were carried out using Chemistry at Harvard Macromolecular Mechanics (CHARMM) Software, version c35b5.^57^ The distance between the centers of mass of the interface residues of the antibody and virus domains is defined as the reaction coordinate. The initial structure of the complex is aligned along the *z*-axis of the local coordinate system, and the virus domain is translated by 0.05 nm increments along the *z* dimension to generate a total of 200 umbrella windows. For both complexes, the largest CoM distance for sampling is around 11 nm. A harmonic restraint with a force constant of 70 kcal/mol·Å^2^ was applied to restrain the relative distance between antibody and virus domain to the target umbrella distance. For each umbrella window, Langevin dynamics simulations were then run at 310 K using a frictional coefficient of 0.050 ps^–1^, an integration time step of 0.015 ps, and the SHAKE algorithm applied to virtual bonds between coarse-grained particles. Exchanges between neighboring windows were attempted every 5000 integration time steps (75 ps). In total, 10 000 exchanges (750 ns of simulation time) were run with the acceptance ratios between neighboring umbrellas between 0.4 and 0.75. The first 1000 attempted exchanges were discarded to allow for equilibration, and the remaining 9000 exchanges were used for analysis.

#### 2.5.4 Determining Dissociation Constant *K*_D_ from REX-US Simulations

We can consider CR3022–RBD and 4A8–NTD complexes as two-body systems and define [A], [B], and [AB] as the respective concentrations of the free monomers and the dimer. For example, with CR3022–RBD, [A] = [CR3022], [B] = [SARS-CoV-2 RBD], and [AB] = [CR3022–RBD]. The simulation results are interpreted under the assumption of a two-state binding model. *P*_b_ is the probability of the system being in the bound states, with *P*_u_ = 1 – *P*_b_ defined as the probability of being in the unbound state. In the unbound state, [A] ≡ [B]. The dissociation constant can be calculated as a function of *P*_b_, *P*_u_, and [A] as^39^
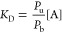
5where the free monomer concentration is defined as
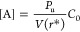
6In eq 6, *C*_0_ = 1660 is the standard concentration used to normalize [A] to the units of molarity. *V*(r*) is the simulation volume in which we found free monomers in the unbound state. As simulations have radial symmetry, r* is the maximum distance between unbound monomers found during the simulation. *P*_b_ is calculated from numerical integration of the potential of mean force (PMF) as
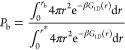
7Here, G_1D_(*r*) is the one-dimensional (1D) PMF constructed from the REX simulations using the weighted histogram analysis method (WHAM) equations,^58^*r*_b_ is the distance threshold separating bound and unbound states, β = 1/*k*_B_*T*, and *r*_b_ is the value of *r* at which the 1D PMF reaches maximum.

## 3 Results and Discussion

### 3.1 CR3022–RBD Is More Stable than 4A8–NTD: Analysis Based on PDB Structures

Using PDB structures with missing residues rebuilt as described in the Section 2, we obtained the CR3022–RBD and 4A8–NTD interfaces shown in Figure S1A1,A2. RBD (chain C) formed contact with both chains A and B of CR3022, while NTD (chain A) interacted with chain B but not with chain C of 4A8. There were more than 42 residues in the CR3022–RBD binding region, while only 27 residues were present at the 4A8–NTD interface.

The network of hydrogen bonds and nonbonded contacts of CR3022–RBD is richer than 4A8–NTD (Figure S1B1,B2). There are 10 and 6 HBs for CR3022–RBD and 4A8–NTD, respectively, while CR3022–RBD has 21 nonbonded contacts, which is more than 14 contacts between 4A8 and NTD. Thus, CR3022 is likely to associate with RBD more strongly than 4A8 with NTD, and this will be confirmed by molecular simulations.

### 3.2 SMD Results

#### 3.2.1 CR3022 Binds to RBD More Strongly than 4A8 to NTD: CHARMM36 FF

**Figure 3:**
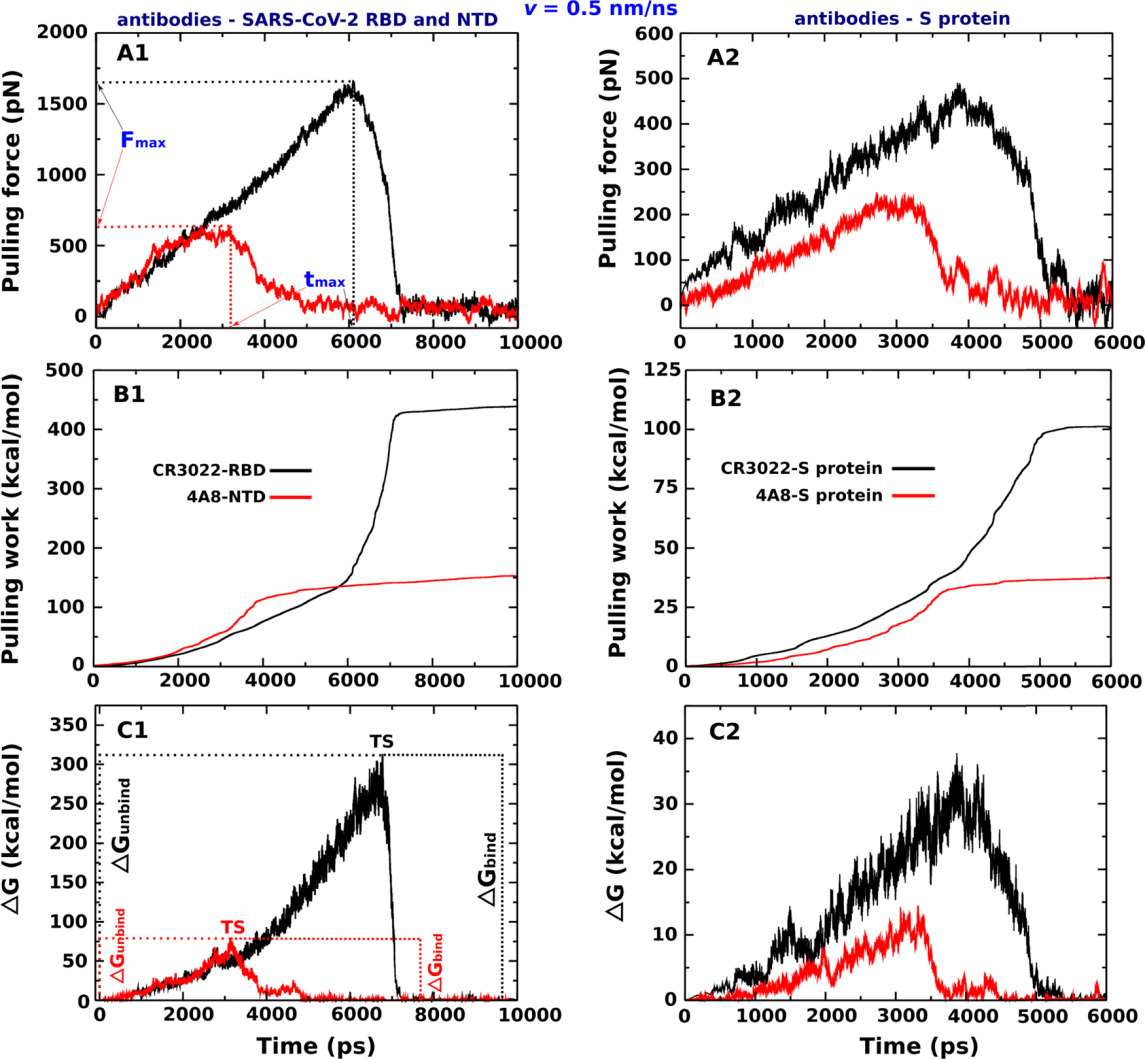
(Left) Pulling force, pulling work, and nonequilibrium energy profiles of CR3022–RBD and 4A8–NTD complexes averaged from five independent SMD runs at *v* = 0.5 nm/ns. (Right) Pulling force, pulling work, and nonequilibrium energy profiles of CR3022–S protein and 4A8–S protein complexes averaged from five independent SMD runs at *v* = 0.5 nm/ns. The results were obtained by the CHARMM36 force field.

We first discuss the results obtained by using the CHARMM36 force field. The force–time profiles of two complexes, obtained at *v* = 0.5 nm/ns (Figure 3) and 1.5 and 5 nm/ns (Figure S2), show that CR3022 binds to RBD more strongly than 4A8 to NTD as the corresponding rupture force *F*_max_ is higher. For *v* = 0.5 nm/ns, *F*_max_ = 1665.2 ± 121.3 and 638.2 ± 57.1 pN for CR3022–RBD and 4A8–NTD, respectively. As expected, the rupture force increases with increasing pulling speed (Table 2).

**Table 2:** Rupture Force (*F*_max_), Unbinding Time (*t*_max_), Work of the External Force (*W*), Nonequilibrium Binding (Δ*G*_bind_), and Unbinding (Δ*G*_unbind_) Free Energy Barriers Obtained from the Five Independent SMD Trajectories of Four Complexes at Pulling Speeds *v* = 0.5, 1.5, and 5 nm/ns^a^

	*F*_max_ (pN)		*t*_max_ (ps)		*W* (kcal/mol)		Δ*G*_bind_(kcal/mol)		Δ*G*_unbind_ (kcal/mol)	
pulling speed *v* (nm/ns)	CR3022–RBD	4A8–NTD	CR3022–RBD	4A8–NTD	CR3022–RBD	4A8–NTD	CR3022–RBD	4A8–NTD	CR3022–RBD	4A8–NTD
0.5	1665.2 ± 121.3	638.2 ± 57.1	6094.0 ± 218.3	2922.0 ± 170.9	438.2 ± 5.9	152.2 ± 4.0	313.5 ± 4.0	79.6 ± 4.1	313.1 ± 4.5	78.4 ± 1.9
1.5	1843.5 ± 146.4	1001.4 ± 85.3	2015.8 ± 131.2	1265.8 ± 154.8	607.2 ± 5.6	305.9 ± 5.7	393.8 ± 5.1	131.1 ± 5.5	387.7 ± 2.6	129.0 ± 5.9
5	2437.5 ± 155.1	1354.8 ± 108.7	726.3 ± 54.8	463.2 ± 57.5	1076.4 ± 7.7	647.7 ± 6.9	498.5 ± 5.6	216.9 ± 7.8	478.9 ± 7.9	189.9 ± 6.7

	CR3022–S protein	4A8- S protein	CR3022- S protein	4A8- S protein	CR3022–S protein	4A8–S protein	CR3022–S protein	4A8–S protein	CR3022–S protein	4A8–S protein
0.5	490.8 ± 36.3	241.4 ± 21.1	3848.0 ± 146.2	2720.0 ± 115.8	100.2 ± 1.0	37.1 ± 1.8	37.8 ± 1.3	14.5 ± 1.2	37.6 ± 1.4	14.3 ± 0.7
1.5	618.0 ± 42.3	559.7 ± 39.8	1030.2 ± 77.9	781.5 ± 74.2	161.2 ± 4.8	111.9 ± 5.8	62.8 ± 3.6	45.4 ± 3.6	62.3 ± 2.8	44.9 ± 2.1
5	947.4 ± 47.7	900.8 ± 48.8	413.2 ± 51.3	324.1 ± 27.7	337.7 ± 4.2	285.9 ± 6.5	88.6 ± 2.6	73.4 ± 3.4	87.3 ± 5.3	70.6 ± 2.3

a The errors represent standard deviations. The results were obtained by the CHARMM36 force field.

Because CR3022 binds to RBD more strongly than 4A8 to NTD, the rupture time *t*_max_ to reach *F*_max_ of the CR3022–RBD complex is also larger than that of 4A8–NTD (Table 2), which is consistent with the results obtained for protein–ligand systems.^39^ The rupture time decreases with an increase of *v*.

The nonequilibrium work *W* was shown to be a better value for characterizing the relative binding affinity than *F*_max_.^59^ It rapidly increased until CR3022 and 4A8 come out from the binding region and reached a stable value when the antibody ceased to interact with the spike protein (Figures 3B and S2). At *v* = 0.5 nm/ns, we obtained *W* = 438.2 ± 5.9 and 152.2 ± 4.0 kcal/mol for CR3022–RBD and 4A8–NTD, respectively (Table 2), which implies that, in agreement with the experiments of Tian et al.^22^ and Chi et al.,^23^ the former complex is more stable than the latter. This also agrees with the data obtained for higher pulling speeds *v* = 1.5 and 5 nm/ns (Table 2).

Using eq 3, we obtained the time dependence of the nonequilibrium binding free energy Δ*G* for two complexes at *v* = 0.5 nm/ns (Figure 3C). The maximum corresponds to the transition state with Δ*G*_TS_. Since at the beginning the state was bound, we have Δ*G*_bound_ = Δ*G*(*t*_0_) ≈ 0 kcal/mol, while the unbound state occurs at the end of simulation^40^ and Δ*G*_unbound_ = Δ*G*(*t*_end_) ≈ 0 kcal/mol. The binding and unbinding free energy barriers, which are defined as ΔΔ*G*_bind_ = Δ*G*_TS_ – Δ*G*_unbound_ and ΔΔ*G*_unbind_ = Δ*G*_TS_ – Δ*G*_bound_, are nearly equal as Δ*G*_unbound_ ≈ Δ*G*_unbound_ ≈ 0.

From Figure 3C, we obtained ΔΔ*G*_unbind_ = 313.1 ± 4.5 and 78.4 ± 1.9 kcal/mol for CR3022–RBD and 4A8–NTD, respectively (see also Table 2), providing additional evidence that CR3022 binds to RBD more tightly than 4A8 to NTD. This conclusion is also valid for other pulling speeds (Table 2 and Figure S2).

#### 3.2.2 CR3022 Binds to RBD More Strongly than 4A8 Binds to NTD: AMBER99SB-DISP FF

To test the robustness of our results against force fields, we additionally performed simulations with the AMBER99SB-DISP force field. The results are shown in Figures S4–S6 and Table S1. *F*_max_, *W*, and ΔΔ*G*_unbind_ obtained for three pulling speeds also support the view that the affinity of binding of CR3022 to RBD is higher than that of 4A8 to NTD.

#### 3.2.3 CR3022 Binds to RBD More Strongly than 4A8 to NTD: Effect of the Entire S Protein Structure

So far, we have considered the interaction of an antibody with either RBD or NTD neglecting the rest of the entire S protein. In this section, we ask whether the remainder of the S protein influences our main conclusion that CR3022 binds to the target more strongly than 4A8. To answer this question, we performed SMD simulations for the CR3022–S protein and 4A8–S protein complexes, as shown in Figure 2B1,B2.

As can be seen from Figures 3 and S6, CR3022 binds to the S protein tighter than 4A8, having higher values of the rupture force, rupture time, pulling work, and unbinding free energy (see also Table 2). However, the interaction of the antibody with the entire S protein is weaker compared to NTD and RBD. For example, at *v* = 0.5 nm/ns, *F*_max_ = 490.8 ± 36.3 and 241.4 ± 21.1 pN for the CR3022–S protein and 4A8–S protein, respectively, while *F*_max_ = 1665.2 ± 121.3 and 638.2 ± 57.1 pN for CR3022–RBD and 4A8–NTD, respectively (Table 2). The same trend was obtained for *W* and ΔΔ*G*_unbind_ at all pulling speeds, while *t*_max_ becomes larger due to weaker interactions (Table 2).

Thus, taking into account the entire structure of protein S changes the absolute binding affinity but leaves the relative binding affinity unchanged. This suggests that CR3022 is a better candidate for the treatment of Covid-19 than 4A8.

#### 3.2.4 Binding of CR3022 and 4A8 to the S Protein Is Driven by Electrostatic Interactions

**Figure 4:**
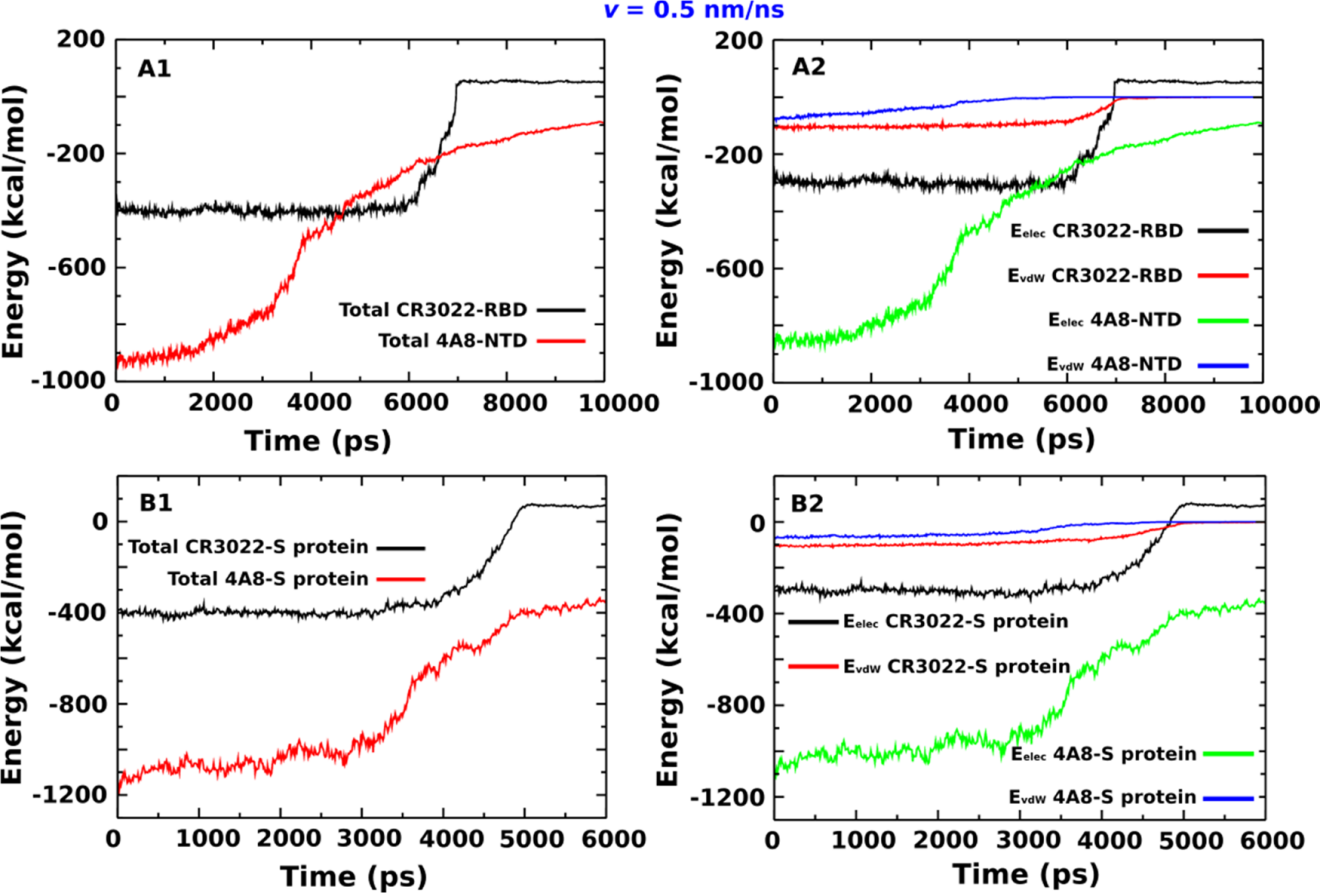
(A1) Total interaction energy (sum of electrostatic and vdW) for the CR3022–RBD and 4A8–NTD complexes. (A2) Same as in (A1) but for the larger CR3022–S protein and 4A8–S protein complexes. (B1) Electrostatic (*E*_elec_) and vdW (*E*_vdW_) interaction energies for the CR3022–RBD and 4A8–NTD complexes. (B2) Same as in (B1) but for larger CR3022–S protein and 4A8–S protein complexes. The results were obtained from five independent SMD runs at *v* = 0.5 nm/ns using the CHARMM36 force field.

The time dependence of vdW, electrostatic, and total (vdW + electrostatic) interaction energies of the CR3022–RBD, 4A8–NTD, CR3022–S protein, and 4A8–S protein complexes, obtained at *v* = 0.5, 1.5, and 5 nm/ns, is shown in Figures 4 and S7. There is a small difference in vdW interaction energies of CR3022–RBD and 4A8–NTD, but a much more pronounced difference is observed for the electrostatic interactions. The same is true for CR3022–S protein and 4A8–S protein complexes. It is important to note that for all complexes, the energy of electrostatic interactions (*E*_elec_) is significantly lower than the vdW energy (*E*_vdW_), which means that their stability is primarily determined by electrostatic interactions.

We calculated the mean interaction energy in the bound state by averaging over the time window [0, *t*_max_]. Note that *t*_max_ depends on the system, *v*, force field, and SMD runs (Tables 2 and S1). At *v* = 0.5 nm/ns for CR3022-RDB, we obtained *E*_elec_ = −299.6 ± 1.4 kcal/mol, which is clearly lower than *E*_vdW_ = −100.6 ± 0.9 kcal/mol (Table 3). A similar result was obtained for CR3022 interacting with the entire S protein with *E*_elec_ = −299.7 ± 1.9 kcal/mol, which is clearly lower than *E*_vdW_ = −96.2 ± 1.4 kcal/mol (Table 3). The difference between the electrostatic and vdW interactions is much more pronounced in the 4A8 case, namely, *E*_elec_ = −817.1 ± 2.4 kcal/mol and *E*_vdW_ = −58.3 ± 0.6 kcal/mol for 4A8–NTD, whereas *E*_elec_ = −1001.9 ± 2.3 kcal/mol and *E*_vdW_ = −61.4 ± 0.3 kcal/mol for 4A8–S protein (Table 3). The dominant role of the electrostatic interactions remains true for other pulling speeds.

**Table 3:** Interaction Energies Obtained by Averaging Five Trajectories of Four Complexes in the Time Window [0 – *t*_max_] at Pulling Speeds *v* = 0.5, 1.5, and 5 nm/ns^a^

	*v* = 0.5 nm/s		*v* = 1.5 nm/s		*v* = 5 nm/s	
interaction energy (kcal/mol)	CR3022–RBD	4A8–NTD	CR3022–RBD	4A8–NTD	CR3022–RBD	4A8–NTD
electrostatic	–299.6 ± 1.4	–817.1 ± 2.4	–296.3 ± 1.2	–820.9 ± 2.3	–287.1 ± 2.5	–830.3 ± 2.2
vdW	–100.6 ± 0.9	–58.3 ± 0.6	–98.4 ± 0.5	–58.9 ± 0.9	–95.6 ± 1.2	–58.6 ± 0.9
total	–400.2 ± 2.3	–875.4 ± 3.0	–394.7 ± 1.7	–879.8 ± 3.2	–382.7 ± 3.7	–888.9 ± 3.1

	CR3022–S protein	4A8–S protein	CR3022–S protein	4A8–S protein	CR3022–S protein	4A8–S protein
electrostatic	–299.7 ± 1.9	–1001.9 ± 2.3	–297.4 ± 1.2	–1043.6 ± 3.2	–290.9 ± 1.7	–1055.7 ± 3.5
vdW	–96.2 ± 1.4	–61.4 ± 0.3	–96.9 ± 0.6	–65.9 ± 0.6	–97.6 ± 0.9	–66.4 ± 1.1
Total	–395.9 ± 3.3	–1063.3 ± 2.6	–394.3 ± 1.8	–1109.5 ± 3.8	–388.5 ± 2.6	–1122.1 ± 4.6

a The errors represent standard deviations.

The importance of electrostatic interactions has also been recognized for the entry of SARS-CoV-2 into cells through binding of the S protein to human ACE2.^39,60^

#### 3.2.5 Charged Residues at the Interface Are Important for Binding Affinity

**Figure 5:**
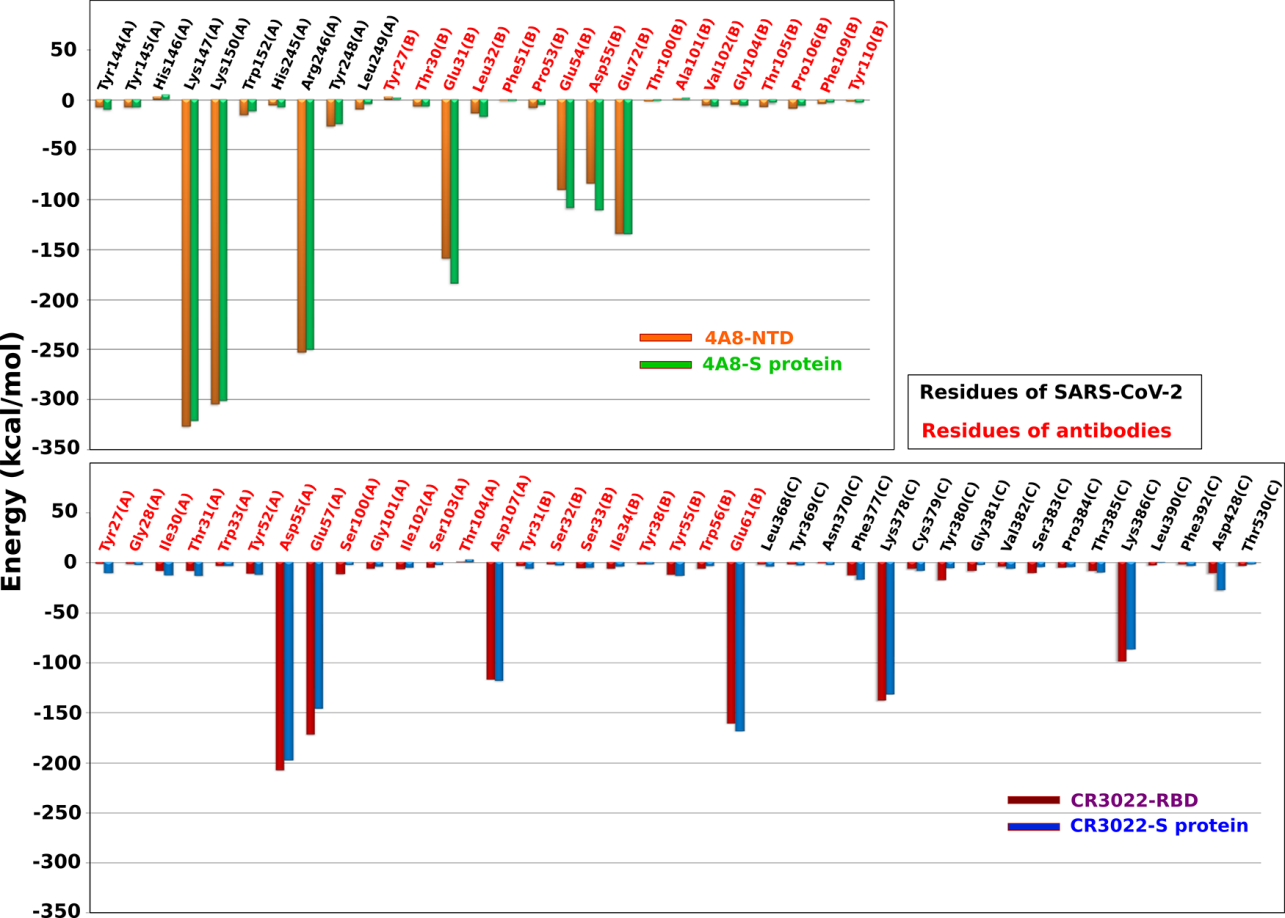
(Top) Total interaction energy of the residues at the binding region (see Figure S2) of the 4A8–NTD and 4A8–S protein complexes. (Bottom) Same as on the top but for the CR3022–RBD and CR3022–S protein complexes. Black and red refer to the residues of SARS-CoV-2 and antibody, respectively. The results were obtained in the time window [0, *t*_max_] at pulling speed *v* = 0.5 nm/ns. The CHARMM36 force field was used.

To understand the role of each residue at the interface in binding of the antibody to the S protein, we calculated the average per-residue interaction energy in the [0, *t*_max_] time window at a pulling speed *v* = 0.5 nm/ns. For both 4A8–NTD and 4A8–S protein complexes, Lys147(A), Lys150(A), and Arg246(A) of the S protein and Glu31(B), Glu54(B), Asp55(B), and Glu72(B) of the 4A8 antibody yielded a significant contribution to the interaction energy (Figure 5, top). In the case of CR3022–RBD and CR3022–S protein complexes (Figure 5, bottom), residues Asp55(A), Glu57(A), Asp107(A), and Glu61(B) of the CR3022 antibody and Lys378(C) and Lys386(C) of the S protein dominate. Importantly, all of the most prominent residues are charged, implying that electrostatic interactions play a dominant role in stabilizing the four complexes studied.

To show that the most important charged residues govern the binding affinity, we carried out simulations where these residues have been replaced by neutral Alanine (Figure S8). For *v* = 0.5 nm/ns and the CHARMM36 force field, these mutations reduced *F*_max_ from 1665.2 ± 121.3 to 768.9 ± 62.6 pN for CR3022–RBD and from 638.2 ± 57.1 to 403.1 ± 51.5 pN for 4A8–NTD, which supports our hypothesis.

### 3.3 Estimation of Dissociation Constant of CR3022–RBD and 4A8–NTD Complexes Using Coarse-Grained Simulations

**Figure 6:**
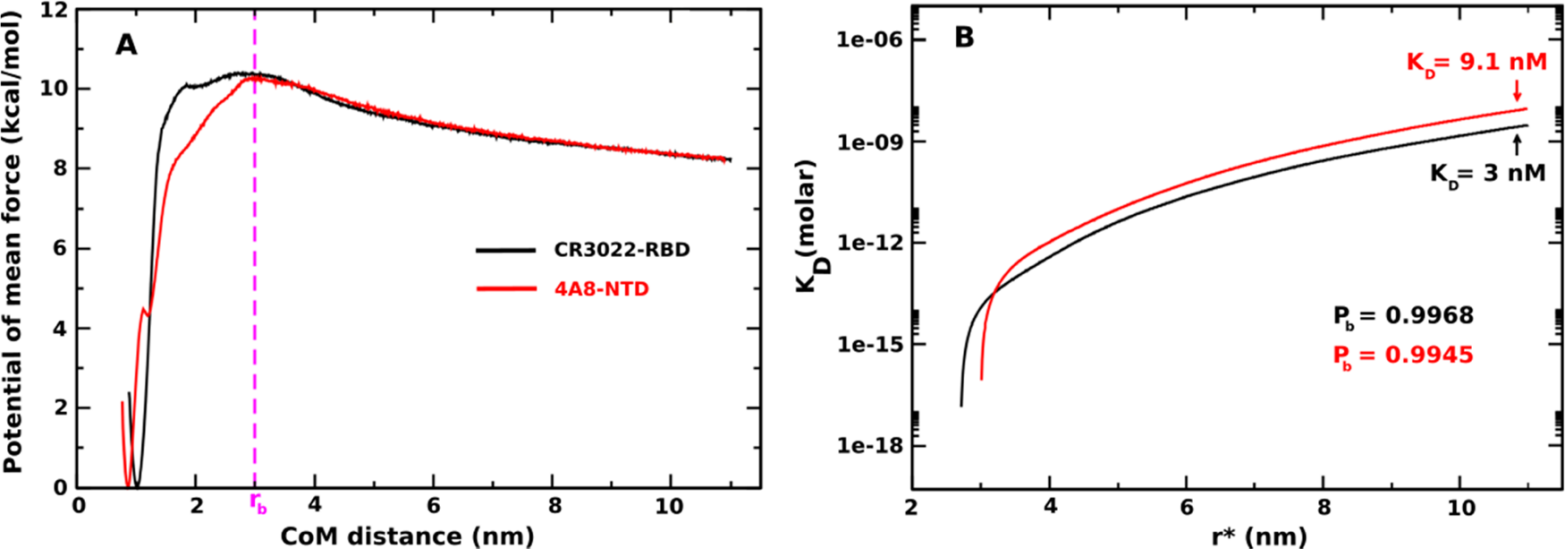
(Left) One-dimensional potential of mean force (1D PMF) of CR3022–RBD (black curve) and 4A8–NTD (red curve). Results were obtained by applying the WHAM analysis for 750 ns REX-US simulations. (Right) *K*_D_ curves as a function of r* corresponding to the change in the total free monomer concentration from eq 5. *P*_b_ and *K*_D_ were determined at r* = 11 nm.

As described in the Section 2, we employed dissociation constant *K*_D_ to evaluate the binding affinity of antibodies to SARS-CoV-2 RBD and SARS-CoV-2 NTD domains. First *P*_b_ is calculated from 1D PMF (eq 7) at different r* values. Figure 6A shows the most stable state locates near the native states with the CoM distance ≈ 1 nm (CR3022–RBD) and ≈0.86 nm (4A8–NTD). The barrier of 1D PMF separating the bound and unbound regimes occurs at ≈3 nm for both complexes so we decided to choose *r*_b_ = 3 nm for the numerical calculation of numerator of eq 7. *P*_u_ = 1 – *P*_b_, and free monomer concentration [A] is calculated using eq 6. Finally, *K*_D_ is obtained from eq 5. Figure 6B plots *K*_D_ curves as a function of r*. As expected, *K*_D_ increases and converges at a large radius r*, which means *K*_D_ physically should not depend on r*. From our simulations, we need to define a cutoff r* corresponding to a total limit volume to define the probability of finding the system in the free monomer state. We determined r* ≈ 11 nm from which there is no longer interaction between the antibody and virus and used this value to calculate *K*_D_.^39^ Our results showed that both antibodies tightly bind to virus domains with *K*_D_ = 9.1 nM for 4A8–NTD and *K*_D_ = 3 nM for CR3022–RBD (Table 1). Thus, from our simulations it can be seen that CR3022 binds to the SARS-CoV-2 RBD domain more strongly than 4A8 binds to SARS-CoV-2 NTD, but the difference is not as great as in SMD (Table 1), because the difference in KD is only about three times. However, it can somehow explain the discrepancy between the results obtained in different experiments. Tian’s group^22^ measured the strong binding affinity of the CR3022–RBD complex with a dissociation constant *K*_D_ = 6.3 nM. Comparing this result with *K*_D_ = 92.7 nM obtained for 4A8–NTD by Chi et al.,^23^ one can conclude that CR3022 binds strongly to SARS-CoV-2 RBD than 4A8 to SARS-CoV-2 NTD. Thus, our results obtained by both all-atom and CG simulations are consistent with these two groups.

It should be noted that for 4A8–NTD our value of *K*_D_ is about an order of magnitude smaller than that of Chi et al.^23^ (Table 1). This level of agreement between simulations and experiments is reasonable when we consider the relationship between *K*_D_ and the binding free energy Δ*G*_bind_ = −*k*_B_*T*  ln(*K*_D_), where *K*_D_ is measured in M. At room temperature, *k*_B_*T* ≈ 0.592 kcal/mol, meaning that a difference in *K*_D_ of one order of magnitude results only in a difference in Δ*G*_bind_ of 1.4 kcal/mol, which is on the order of the calculation error.

Experimentally, Yuan’s group^21^ pointed out that CR3022 has a weak neutralizing effect for SARS-COV-2, as it has a low binding affinity to RBD with *K*_D_ = 115 nM (Table 1). This result is in conflict with that of Tian et al.,^22^ who reported a lower value of *K*_D_.

One advantage of our computational study is that we studied two complexes using the same model, while experiments conducted by different groups were carried out under different conditions, making it difficult to directly compare experimental results. Our simulations showed that CR3022 binds to RBD more strongly than 4A8 to NTD, which is consistent with Tian et al.^22^ and Chi et al.^23^ but not with Yuan et al.^21^ (Table 1). From our computational point of view, the fact that the result of Yuan et al.^21^ contradicts that of Tian et al.^22^ is explained by the different conditions used in their in vitro experiments, namely, CR3022 was expressed in mammalian cells in Yuan et al.^21^ but in *Escherichia coli* in Tian et al.^22^ Moreover, SARS-CoV-2 RBD was obtained from insect cells in Yuan et al.^21^ but from mammalian cells in Tian et al.^22^ Since *K*_D_ obtained in our CG simulations is close to that of Tian et al.,^22^ our model can be expected to reasonably capture the conditions used in this group’s experiment. A modification of our models to mimic the experimental conditions in Yuan et al.^21^ is nontrivial and requires further study.

## 4 Conclusions

In this work, we applied all-atom SMD and coarse-grained simulations to study the binding affinity of CR3022 and 4A8 antibodies to the S protein of SARS-CoV-2. SMD simulations showed that CR3022 displays a higher binding propensity to RBD than 4A8 to NTD, which is consistent with the result obtained by coarse-grained REX-US simulations that the dissociation constant *K*_D_ of CR3022–RBD is approximately three times smaller than that of 4A8–NTD. Our results are in good agreement with the experimental data of Tian et al.^22^ and Chi et al.,^23^ but they are in contrast to the experimental results of Yuan et al.^21^ The contribution of electrostatic interactions to the stability of four complexes, including CR3022–RBD, 4A8–NTD, CR3022–S protein, and 4A8–S protein, is more significant compared to vdW interactions. In terms of binding capability, CR3022 is a better candidate for Covid-19 treatment than 4A8.

Since the RBD and NTD binding sites contain charged residues, electrostatic interactions are likely to play an important role not only in CR3022 and 4A8 binding but also in other antibodies. Our preliminary results on the binding of antibodies REGN10933 and REGN10987,^61^ as well as nanobodies H11-H4,^62^ support this hypothesis, but more systems need to be examined to arrive at a firm conclusion.

Our prediction that electrostatic interactions play a key role in the binding of antibodies to SARS-CoV-2 could open up a new strategy for developing effective antibodies against Covid-19. For example, good candidates should contain many charged amino acids in the region that binds to the spike protein. Moreover, since the important residues of the spike protein are positively charged (Lys and Arg, Figure 5), potential antibodies must have negatively charged residues (Asp and Glu). From a methodological point of view, it is important to emphasize that coarse-grained models in combination with REX-US provide a reasonable tool for estimating the dissociation constant of two proteins.

## Supporting Information Available

Networks of hydrogen-bonded and nonbonded contacts of CR3022–RBD and 4A8–NTD complexes (Figure S1); time dependence of the pulling force, work, and nonequilibrium free energy of CR3022–RBD and 4A8–NTD complexes (Figure S2); pulling force–time profiles of CR3022–RBD and 4A8–NTD complexes (Figure S3); work–time profiles of CR3022–RBD and 4A8–NTD complexes (Figure S4); time dependence of the nonequilibrium free energy of CR3022–RBD and 4A8–NTD (Figure S5); time dependence of the pulling force, work, and nonequilibrium free energy of CR3022–S protein and 4A8–S protein complexes (Figure S6); time dependence of the electrostatic and vdW interaction energies for four complexes (Figure S7); time dependence of the pulling force for the CR3022–RBD and 4A8–NTD complexes (Figure S8); rupture force (*F*_max_), unbinding time (*t*_max_), work of external force (*W*), nonequilibrium binding (Δ*G*_bind_), and unbinding (Δ*G*_unbind_) free energy barriers of both CR3022–RBD and 4A8–NTD complexes (Table S1) (PDF)
SI-JPCB (ZIP)

## Notes

The input and output files of our SMD and coarse-grained simulations are available in the “Data_share.zip” file that includes ***All-atom simulation data***: *SMD Inputs and Outputs*: The input files and final output coordinates of SMD simulations are provided in the “SMD_data” folder. Subfolder “INPUT” of “SMD_structure_data” contains the initial files (.pdb) for performing SMD simulations. Subfolder “OUTPUT” of “SMD_structure_data” contains the last structures (.gro) of SMD simulations for only one trajectory. *Raw data for figures* The subfolder “SMD_draw_data” of “SMD_data” contains files.dat and.xvg forplotting the pulling force, pulling work, nonequilibrium energy, and interaction energy. ***Coarse-grained simulation data:****Inputs and Outputs for REX-US simulations*: The input files and final output coordinates of 200 umbrella windows of REX-US simulations are provided as additional files in the folder “REX-US_simulation_data”. Subfolder INPUT contains files.cor,.prm,.top,.psf, and 200 initial structures of 200 umbrella windows. Subfolder OUTPUT contains the last structures at 750 ns (.cor files) of 200 umbrella windows. *Raw data for figures:* The folder “Draw_data” includes subfolders “Figure_6A” and “Figure_6B”. Each of these contains files.dat for plotting the potential of mean forces (Figure 6A) and *K*_D_ curves (Figure 6B).

## References

[ref1] Huang C.; Wang Y.; Li X.; et al. Clinical features of patients infected with 2019 novel coronavirus in Wuhan, China. Lancet 2020, 395, 497–506. 10.1016/S0140-6736(20)30183-5. 31986264 PMC7159299

[ref2] Gorbalenya A.; Baker S.; Baric R.; et al. The species severe acute respiratory syndrome-related coronavirus: Classifying 2019-nCoV and naming it SARS-CoV-2. Nat. Microbiol. 2020, 5, 536–544. 10.1038/s41564-020-0695-z. 32123347 PMC7095448

[ref3] Wang Y.; Dingyu Z.; et al. Remdesivir in adults with severe COVID-19: a randomised, double-blind, placebo-contolled, multicentre trial. Lancet 2020, 395, 1569–1578. 10.1016/S0140-6736(20)31022-9. 32423584 PMC7190303

[ref4] Horby P.; Lim W. S.; Emberson J.; et al. Dexamethasone in hospitalized patients with Covid-19 - Preliminary Report. N. Engl. J. Med. 2021, 384, 693–704. 10.1056/NEJMoa2021436. 32678530 PMC7383595

[ref5] Khamsi R. Rogue abntibodies could be driving severe COVID-19. Nature 2021, 590, 29–31. 10.1038/d41586-021-00149-1. 33469204

[ref6] Jon C. South Africa suspends use of AstraZeneca’s COVID-19 vaccine after it fails to clearly stop virus variant. Science 2021, https://www.sciencemag.org/news/2021/02/south-africa-suspends-use-astrazenecas-covid-19-vaccine-after-it-fails-clearly-stop.

[ref7] Jiang S.; Hillyer C.; Du L. Neutralizing antibodies against SARS-CoV-2 and other human Coronaviruses. Trends Immunol. 2020, 41, 355–359. 10.1016/j.it.2020.03.007. 32249063 PMC7129017

[ref8] Tortorici M. A.; Weesler D. Advances in Virus Research; Rey F. A., Ed.; Academic Press: 2019; Vol. 105, Chapter 4, pp 93–116. 10.1016/bs.aivir.2019.08.002 PMC7112261 31522710

[ref9] Simmons G.; Zmora P.; Gierer S.; et al. Proteolytic activation of the SARS-coronavirus Spike protein: Cutting enzymes at the cutting edge of antiviral research. Antiviral Res. 2013, 100, 605–614. 10.1016/j.antiviral.2013.09.028. 24121034 PMC3889862

[ref10] Gallagher T. M.; Buchmeier M. J. Coronavirus Spike proteins in viral entry and pathogenesis. Virology 2001, 279, 371–374. 10.1006/viro.2000.0757. 11162792 PMC7133764

[ref11] Belouzard S.; Chu V. C.; Whittaker G. R. Activation of the SARS coronavirus Spike protein via sequential proteolytic cleavage at two distinct sites. Proc. Natl. Acad. Sci. U.S.A. 2009, 106, 5871–5876. 10.1073/pnas.0809524106. 19321428 PMC2660061

[ref12] Wrapp D.; Wang N.; Corbett K. S.; et al. Cryo-EM structure of the 2019-nCoV Spike in the prefusion conformation. Science 2020, 367, 1260–1263. 10.1126/science.abb2507. 32075877 PMC7164637

[ref13] Walls A. C.; Park Y.-J.; Tortorici M. A.; et al. Structure, function, and antigenicity of the SARS-CoV-2 Spike glycoprotein. Cell 2020, 180, 281–292. 10.1016/j.cell.2020.02.058. PMC7102599 32155444

[ref14] Krempl C.; Schultze B.; Laude H.; et al. Point mutations in the S protein connect the sialic acid binding activity with the enteropathogenicity of transmissible gastroenteritis coronavirus. J. Virol. 1997, 71, 3285–3287. 10.1128/jvi.71.4.3285-3287.1997. 9060696 PMC191465

[ref15] Künkel F.; Herrler G. Structural and functional analysis of the S proteins of two human coronavirus OC43 strains adapted to growth in different cells. Arch. Virol. 1996, 141, 1123–1131. 10.1007/BF01718615. 8712929 PMC7086633

[ref16] Lu G.; Wang Q.; Gao G. F. Bat-to-human: Spike features determining ‘host jump’ of coronaviruses SARS-CoV, MERS-CoV, and beyond. Trends Microbiol. 2015, 23, 468–478. 10.1016/j.tim.2015.06.003. 26206723 PMC7125587

[ref17] Zhou H.; Chen Y.; Zhang S.; et al. Structural definition of a neutralization epitope on the N-terminal domain of MERS-CoV Spike glycoprotein. Nat. Commun. 2019, 10, 3068 10.1038/s41467-019-10897-4. 31296843 PMC6624210

[ref18] Tai W.; He L.; Zhang X.; et al. Characterization of the receptor-binding domain (RBD) of 2019 novel coronavirus: implication for development of RBD protein as a viral attachment inhibitor and vaccine. Cell. Mol. Immunol. 2020, 17, 613–620. 10.1038/s41423-020-0400-4. 32203189 PMC7091888

[ref19] Lan J.; Ge J.; Yu J.; et al. Structure of the SARS-CoV-2 Spike receptor-binding domain bound to the ACE2 receptor. Nature 2020, 581, 215–220. 10.1038/s41586-020-2180-5. 32225176

[ref20] ter Meulen J.; van den Brink E. N.; Poon L. L. M.; et al. Human monoclonal antibody combination against SARS coronavirus: Synergy and coverage of escape mutants. PLoS Med. 2006, 3, e237 10.1371/journal.pmed.0030237. 16796401 PMC1483912

[ref21] Yuan M.; Wu N. C.; Zhu X.; et al. A highly conserved cryptic epitope in the receptor binding domains of SARS-CoV-2 and SARS-CoV. Science 2020, 368, 630–633. 10.1126/science.abb7269. 32245784 PMC7164391

[ref22] Tian X.; Li C.; Huang A.; et al. Potent binding of 2019 novel coronavirus Spike protein by a SARS coronavirus-specific human monoclonal antibody. Emerg. Microbes Infect. 2020, 9, 382–385. 10.1080/22221751.2020.1729069. 32065055 PMC7048180

[ref23] Chi X.; Yan R.; Zhang J.; et al. A neutralizing human antibody binds to the N-terminal domain of the Spike protein of SARS-CoV-2. Science 2020, 369, 650–655. 10.1126/science.abc6952. 32571838 PMC7319273

[ref24] Vajda S.; Yueh C.; et al. New additions to the ClusPro server motivated by CAPRI. Proteins 2017, 85, 435–444. 10.1002/prot.25219. 27936493 PMC5313348

[ref25] Kozakov D.; Hall D. R.; et al. The ClusPro web server for protein-protein docking. Nat. Protoc. 2017, 12, 255–278. 10.1038/nprot.2016.169. 28079879 PMC5540229

[ref26] Webb B.; Sali A. Comparative protein structure modeling using MODELLER. Curr. Protoc. Bioinf. 2016, 54, 5.6.1–5.6.37. 10.1002/cpbi.3. PMC5031415 27322406

[ref27] Huang J.; MacKerell A. D. Jr. CHARMM36 all-atom additive protein force field: Validation based on comparison to NMR data. J. Comput. Chem. 2013, 34, 2135–2145. 10.1002/jcc.23354. 23832629 PMC3800559

[ref28] Robustelli P.; Piana S.; Shaw D. E. Developing a molecular dynamics force field for both folded and disordered protein states. Proc. Natl. Acad. Sci. U.S.A. 2018, 115, E4758–E4766. 10.1073/pnas.1800690115. 29735687 PMC6003505

[ref29] Abraham M. J.; Murtola T.; Schulz R.; et al. GROMACS: High performance molecular simulations through multi-level parallelism from laptops to supercomputers. SoftwareX 2015, 1-2, 19–25. 10.1016/j.softx.2015.06.001.

[ref30] Bussi G.; Donadio D.; Parrinello M. Canonical sampling through velocity rescaling. J. Chem. Phys. 2007, 126, 014101 10.1063/1.2408420. 17212484

[ref31] Parrinello M.; et al. Polymorphic transitions in single crystals: A new molecular dynamics method. J. Appl. Phys. 1981, 52, 7182 10.1063/1.328693.

[ref32] Jorgensen W. L.; Jenson C. Temperature dependence of TIP3P, SPC, and TIP4P water from NPT Monte Carlo simulations: Seeking temperatures of maximum density. J. Comput. Chem. 1998, 19, 1179–1186. 10.1002/(SICI)1096-987X(19980730)19:10<1179::AID-JCC6>3.0.CO;2-J.

[ref33] Hess B.; Bekker H.; Berendsen H. J. C.; et al. LINCS: A linear constraint solver for molecular simulations. J. Comput. Chem. 1997, 18, 1463–1472. 10.1002/(SICI)1096-987X(199709)18:12<1463::AID-JCC4>3.0.CO;2-H.

[ref34] Darden T.; York D.; Pedersen L. Particle mesh Ewald: An Nlog(N) method for Ewald sums in large systems. J. Chem. Phys. 1993, 98, 10089 10.1063/1.464397.

[ref35] Hockney R. W.; Goel S. P.; Eastwood J. W. Quiet high-resolution computer models of a plasma. J. Comput. Phys. 1974, 14, 148–158. 10.1016/0021-9991(74)90010-2.

[ref36] Nguyen H.; Do N.; Phan T.; et al. Steered molecular dynamics for investigating the interactions between Insulin Receptor Tyrosine Kinase (IRK) and variants pf Protein Tyrosine Phosphatase 1B (PTP1B). Appl. Biochem. Biotechnol. 2018, 184, 401–413. 10.1007/s12010-017-2549-6. 28707052

[ref37] Nguyen H.; Nguyen H. L.; Linh H. Q.; et al. Binding affinity of the L-742,001 inhibitor to the endonuclease domain of A/H1N1/PA influenza virus variants: Molecular simulation approaches. Chem. Phys. 2018, 500, 26–36. 10.1016/j.chemphys.2017.11.005.

[ref38] Pham T.; Nguyen H. L.; Phan-Toai T.; et al. Investigation of binding affinity between potential antiviral agents and PB2 protein of influenza A: Non-equilibrium molecular dynamics simulation approach. Int. J. Med. Sci. 2020, 17, 2031–2039. 10.7150/ijms.46231. 32788882 PMC7415388

[ref39] Nguyen H. L.; Lan P. D.; Thai N. Q.; et al. Does SARS-CoV-2 bind to human ACE2 more strongly than does SARS-CoV?. J. Phys. Chem. B. 2020, 124, 7336–7347. 10.1021/acs.jpcb.0c04511. 32790406 PMC7433338

[ref40] Truong D. T.; Li M. S. Probing the binding affinity by Jarzynski’s nonequilibrium binding free energy and rupture time. J. Phys. Chem. B 2018, 122, 4693–4699. 10.1021/acs.jpcb.8b02137. 29630379

[ref41] Nguyen H.; Le L. Steered molecular dynamics approach for promising drugs for influenza A virus targeting M2 channel proteins. Eur. Biophys. J. 2015, 44, 447–455. 10.1007/s00249-015-1047-4. 26033540

[ref42] Li M. S.; Mai B. K. Steered molecular dynamics-A promising tool for drug design. Curr. Bioinf. 2012, 7, 342–351. 10.2174/157489312803901009.

[ref43] The PyMOL Molecular Graphics System. version 2.0 Schrödinger, LLC.

[ref44] Binnig G.; Quate C. F.; et al. Atomic force microscope. Phys. Rev. Lett. 1986, 56, 930 10.1103/PhysRevLett.56.930. 10033323

[ref45] Jarzynski C. Nonequilibrium equality for free energy differences. Phys. Rev. Lett. 1997, 78, 2690 10.1103/PhysRevLett.78.2690.

[ref46] Hummer G.; Szabo A. Free energy reconstruction from nonequilibrium single-molecule pulling experiments. Proc. Natl. Acad. Sci. U.S.A. 2001, 98, 3658–3661. 10.1073/pnas.071034098. 11274384 PMC31107

[ref47] Wallace A. C.; Laskowski R. A.; Thornton J. M. LIGPLOT: A program to generate schematic diagrams of protein-ligand interactions. Protein Eng., Des. Sel. 1995, 8, 127–134. 10.1093/protein/8.2.127. 7630882

[ref48] Leininger S. E.; Trovato F.; et al. Domain topology, stability, and translation speed determine mechanical force generation on the ribosome. Proc. Natl. Acad. Sci. U.S.A. 2019, 116, 5523–5532. 10.1073/pnas.1813003116. 30824598 PMC6431206

[ref49] Karanicolas J.; Brooks C. The origins of asymmetry in the folding transition states of protein L and protein G. Protein Sci. 2002, 11, 2351–2361. 10.1110/ps.0205402. 12237457 PMC2373711

[ref50] Best R. B.; Chen Y. G.; Hummer G. Slow protein conformational dynamics from multiple experimental structures: The helix/sheet transition of Arc repressor. Structure 2005, 13, 1755–1763. 10.1016/j.str.2005.08.009. 16338404

[ref51] O’Brien E. P.; Ziv G.; Haran G.; Brooks B. R.; Thirumalai D. Effects of denaturants and osmolytes on proteins are accurately predicted by the molecular transfer model. Proc. Natl. Acad. Sci. U.S.A. 2008, 105, 13403–13408. 10.1073/pnas.0802113105. 18757747 PMC2533202

[ref52] O’Brien E. D.; Christodoulou J.; et al. Trigger factor slows co-translational folding through kinetic trapping while sterically protecting the nascent chain from aberrant cytosolic interactions. J. Am. Chem. Soc. 2012, 134, 10920–10932. 10.1021/ja302305u. 22680285

[ref53] Karanicolas J.; Brooks C. L. 3rd. Improsved Go-like models demonstrate the robustness of protein folding mechanisms towards non-native interactions. J. Mol. Biol. 2003, 334, 309–325. 10.1016/j.jmb.2003.09.047. 14607121

[ref54] Heinig M.; Frishman D. STRIDE: a web server for secondary structure assignment from known atomic coordinates of proteins. Nucl. Acid. Res. 2004, 32, W500–W502. 10.1093/nar/gkh429. PMC441567 15215436

[ref55] Betancourt M. R.; Thirumalai D. Pair potentials for protein folding: Choice of reference states and sensitivity of predicted native states to variations in the interaction schemes. Protein Sci. 1999, 8, 361–369. 10.1110/ps.8.2.361. 10048329 PMC2144252

[ref56] Nissley D. A.; Vu Q. V.; et al. Electrostatic interactions govern extreme nascent protein ejection times from ribosomes and can delay ribosome recycling. J. Am. Chem. Soc. 2020, 142, 6103–6110. 10.1021/jacs.9b12264. 32138505 PMC7312765

[ref57] Brooks B. R.; Brooks C. L. III; et al. CHARMM: The biomolecular simulation program. J. Comput. Chem. 2009, 30, 1545–1614. 10.1002/jcc.21287. 19444816 PMC2810661

[ref58] Kumar S.; Rosenberg J. M.; et al. The weighted histogram analysis method for free- energy calculations on biomolecules. I. The method. J. Comput. Chem. 1992, 13, 1011–1021. 10.1002/jcc.540130812.

[ref59] Vuong Q. V.; Tin T. T.; Mai S. L. A new method for navigating optimal direction for pulling ligand from binding pocket: Application to ranking binding affinity by steered molecular dynamics. J. Chem. Inf. Model. 2015, 55, 2731–2738. 10.1021/acs.jcim.5b00386. 26595261

[ref60] Piotr H. P. Charged amino acids may promote coronavirus SARS-CoV-2 fusion with the host cell. AIMS. Biophysics 2020, 8, 111–120.

[ref61] Hansen J.; Alina B.; et al. Studies in humanized mice and convalescent humans yield a SARS-CoV-2 antibody cocktail. Science 2020, 369, 1010–1014. 10.1126/science.abd0827. 32540901 PMC7299284

[ref62] Huo J.; Audrey L. B.; et al. Neutralizing nanobodies bind SARS-CoV-2 spike RBD and block interaction with ACE2. Nat. Struct. Mol. Biol. 2020, 27, 846–854. 10.1038/s41594-020-0469-6. 32661423

