# Supplementary material for: Electrostatic Interactions Explain the Higher Binding Affinity of the CR3022 Antibody for SARS-CoV-2 than the 4A8 Antibody
Source: J Phys Chem B. 2021 Jul 6;125(27):7368–79. doi: 10.1021/acs.jpcb.1c03639 (PMC8276604; doi:10.1021/acs.jpcb.1c03639)
Supplement: Supplementary file 1 — jp1c03639_si_001.pdf [file jp1c03639_si_001.pdf]

Supporting information

Electrostatic interactions explain the higher binding affinity of CR3022 antibody for SARS-CoV-2 over the 4A8 antibody

Hung Nguyen[1,+], Pham Dang Lan[2,3,+], Daniel A. Nissley[4], Edward P. O'Brien[5, 6, 7], and Mai Suan Li[1,*]

[1]Institute of Physics, Polish Academy of Sciences, al. Lotnikow 32/46, 02-668, Warsaw, Poland

[2]Life Science Lab, Institute for Computational Science and Technology, Quang Trung Software City, Tan Chanh Hiep Ward, District 12, Ho Chi Minh City, Vietnam

[3]Faculty of Physics and Engineering Physics, VNUHCM-University of Science, 227, Nguyen Van Cu Street, District 5, Ho Chi Minh City, Vietnam

[4]Department of Statistics, University of Oxford, Oxford, United Kingdom

[5]Department of Chemistry, Penn State University, University Park, Pennsylvania, United States

[6]Bioinformatics and Genomics Graduate Program, The Huck Institutes of the Life Sciences, Penn State University, University Park, Pennsylvania, United States

[7]Institute for Computational and Data Sciences, Penn State University, University Park,

[+]These authors contributed equally

*Email: masli@ifpan.edu.pl

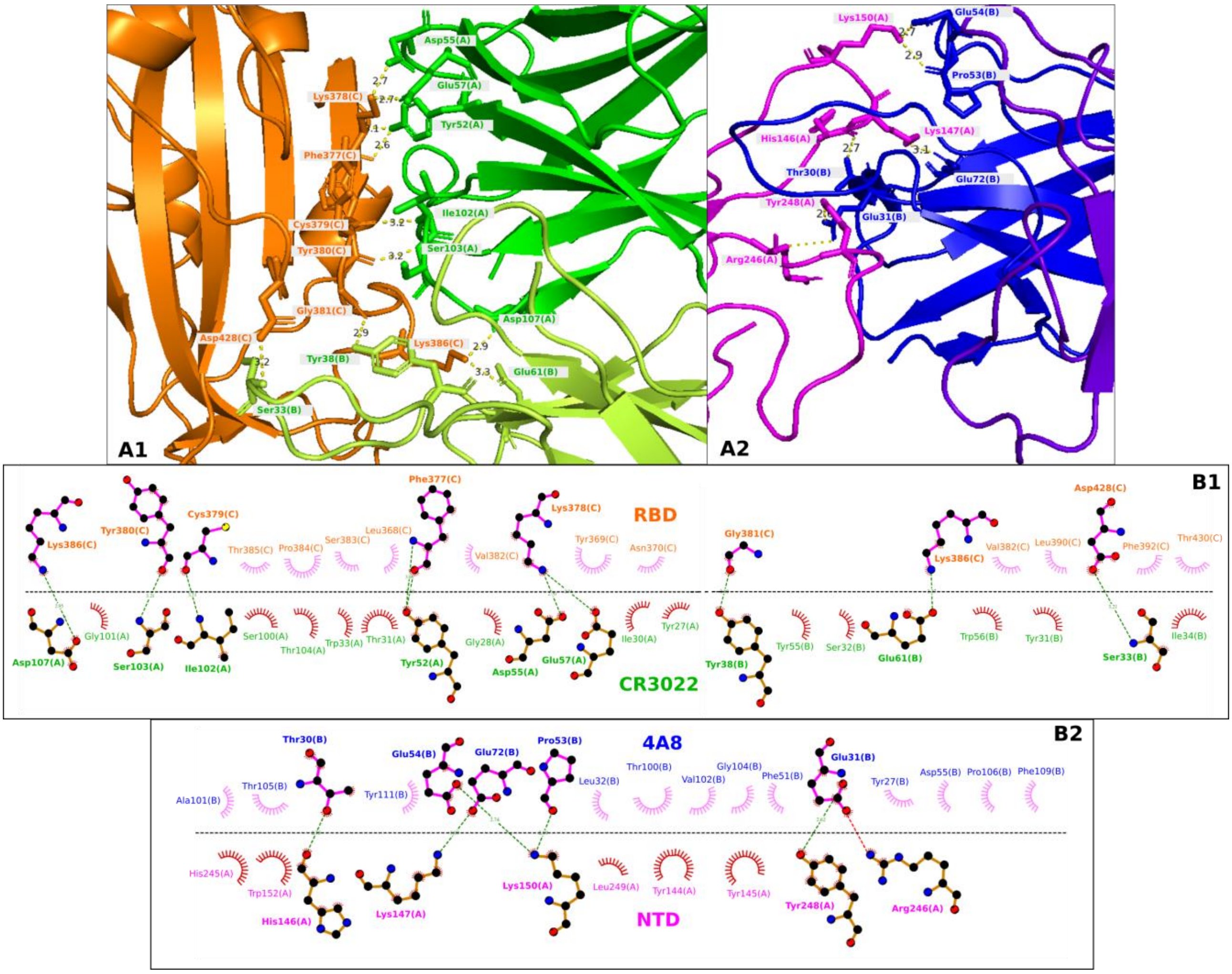

Figure S1. (A1) The interface between RBD (chain C, orange) and CR3022 (chains A and B, green and lemon). (A2) The same as in A1 but for the complex of NTD (chain A, magenta) and 4A8 (chain B and C, blue and purple blue). Networks of hydrogen bond and non-bonded contacts of CR3022-RBD (B1) and 4A8-NTD (B2) complexes. Dotted line refers to HB, while the "eye" refers to NBC.

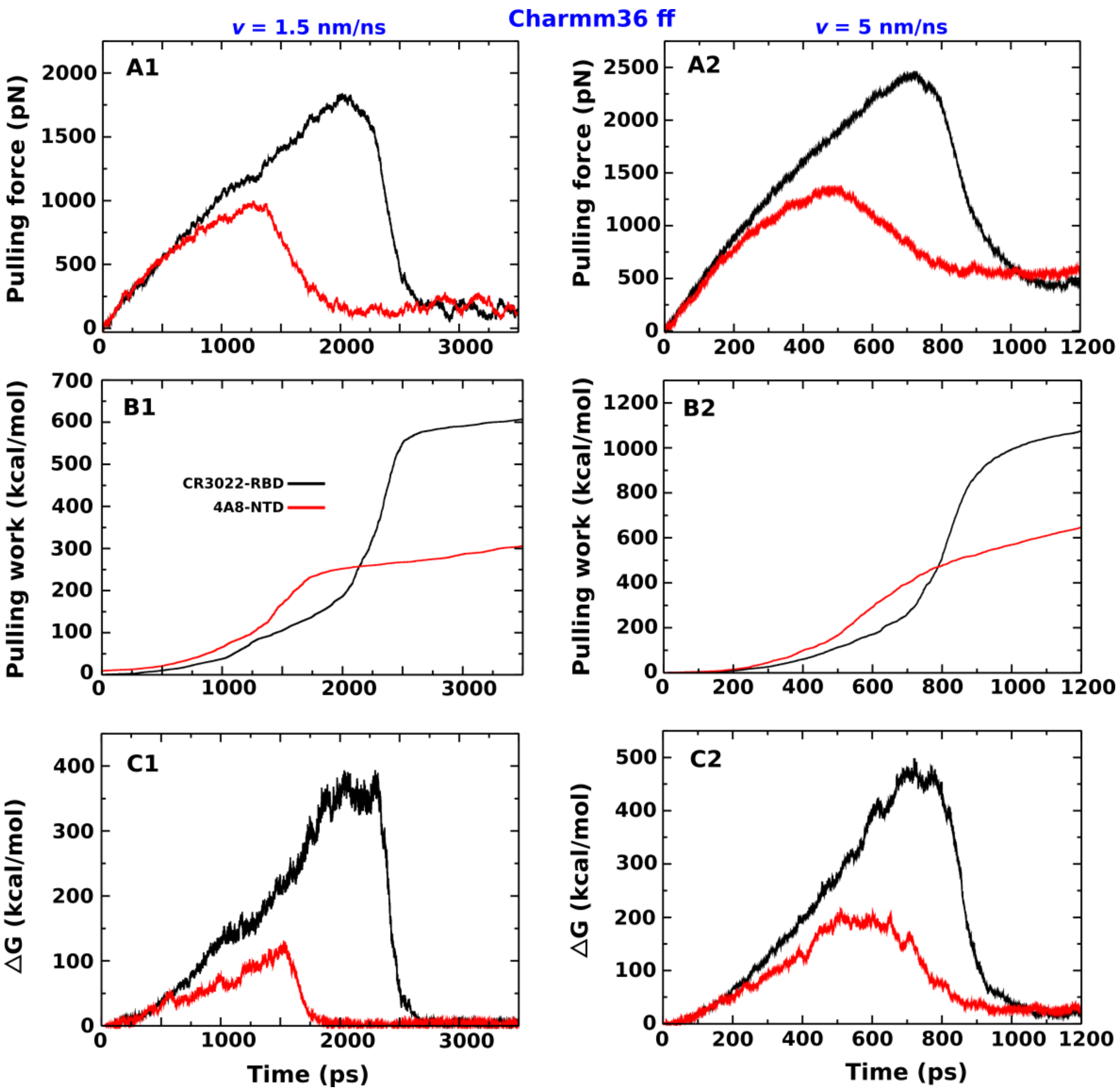

Figure S2. The time dependence of the pulling force, work and non-equilibrium free energy of CR3022-RBD and 4A8-NTD complexes. The results averaged over five independent SMD runs at v = 1.5 (left) and v = 5 nm/ns (right). The CHARMM 36 force field was used.

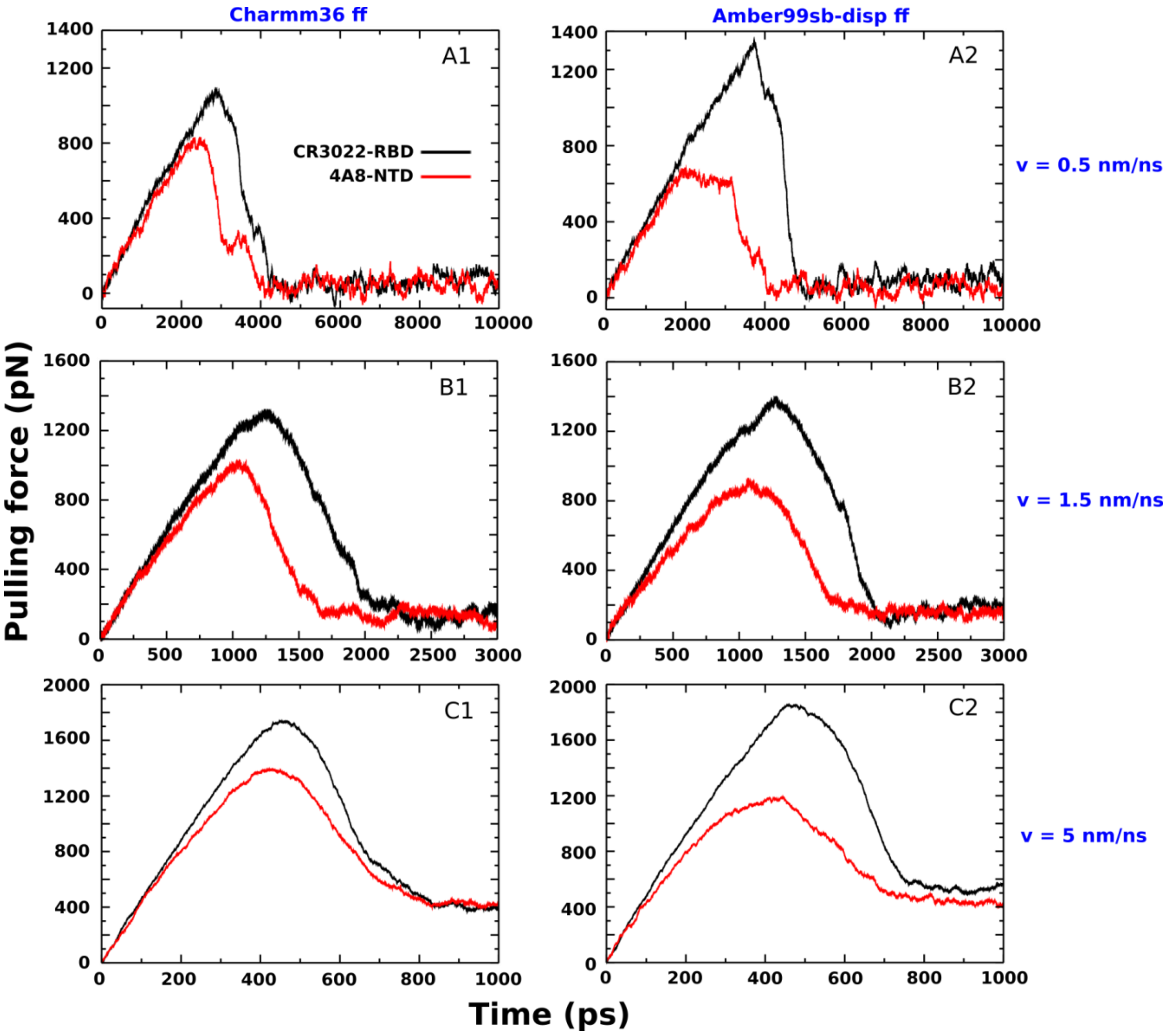

Figure S3. The pulling force-time profiles of CR3022-RBD and 4A8-NTD complexes at *v* = 0.5, *v* = 1.5 and *v* = 5 nm/ns for CHARMM 36 (left) and AMBER99SB-DISP (right) force fields. The results were obtained from five independent SMD runs.

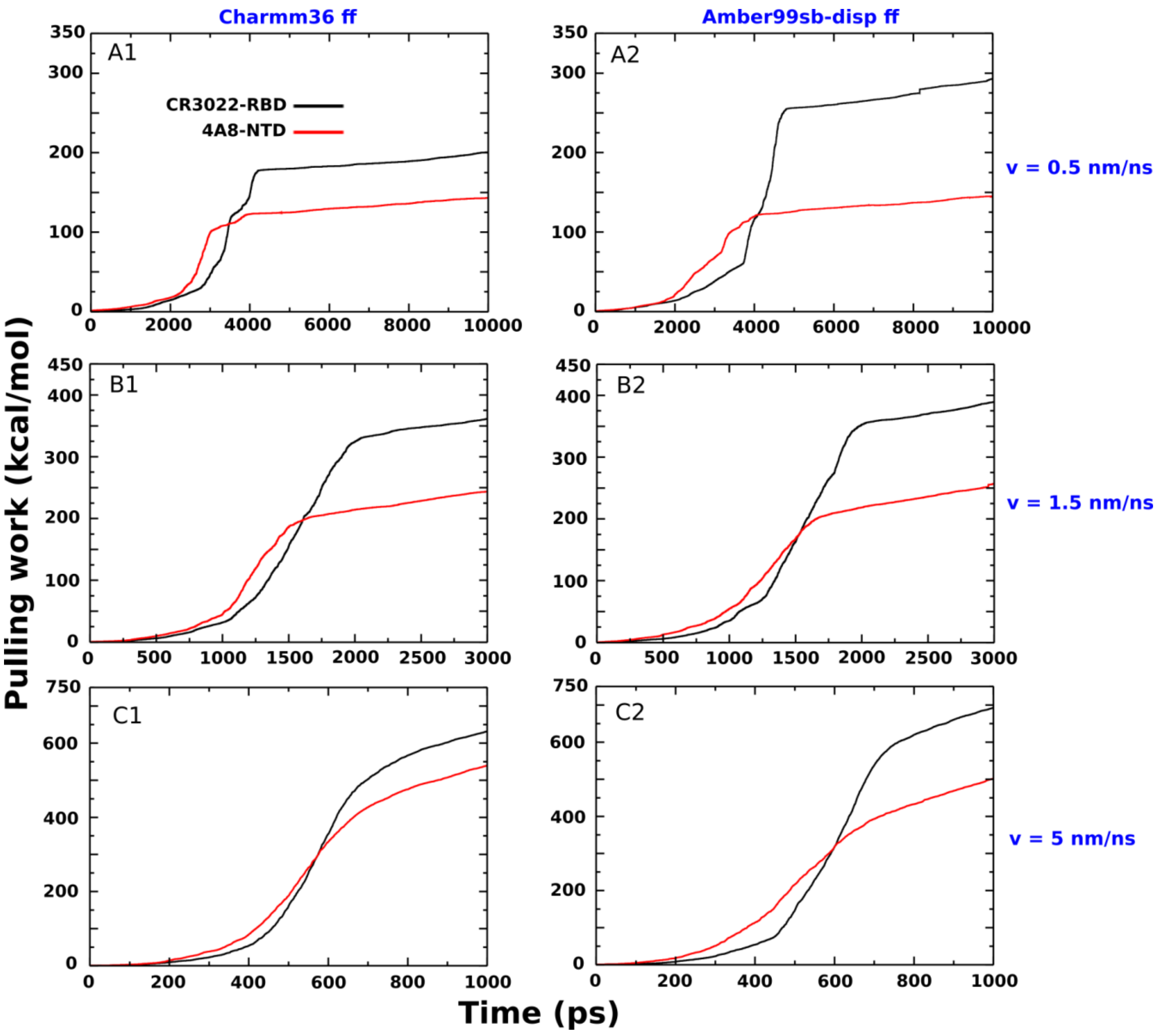

Figure S4. Work-time profiles of CR3022-RBD and 4A8-NTD complexes at *v* = 0.5, *v* = 1.5 and *v* = 5 nm/ns for CHARMM 36 (left) and AMBER99SB-DISP (right) force fields. The results were obtained from five independent SMD runs.

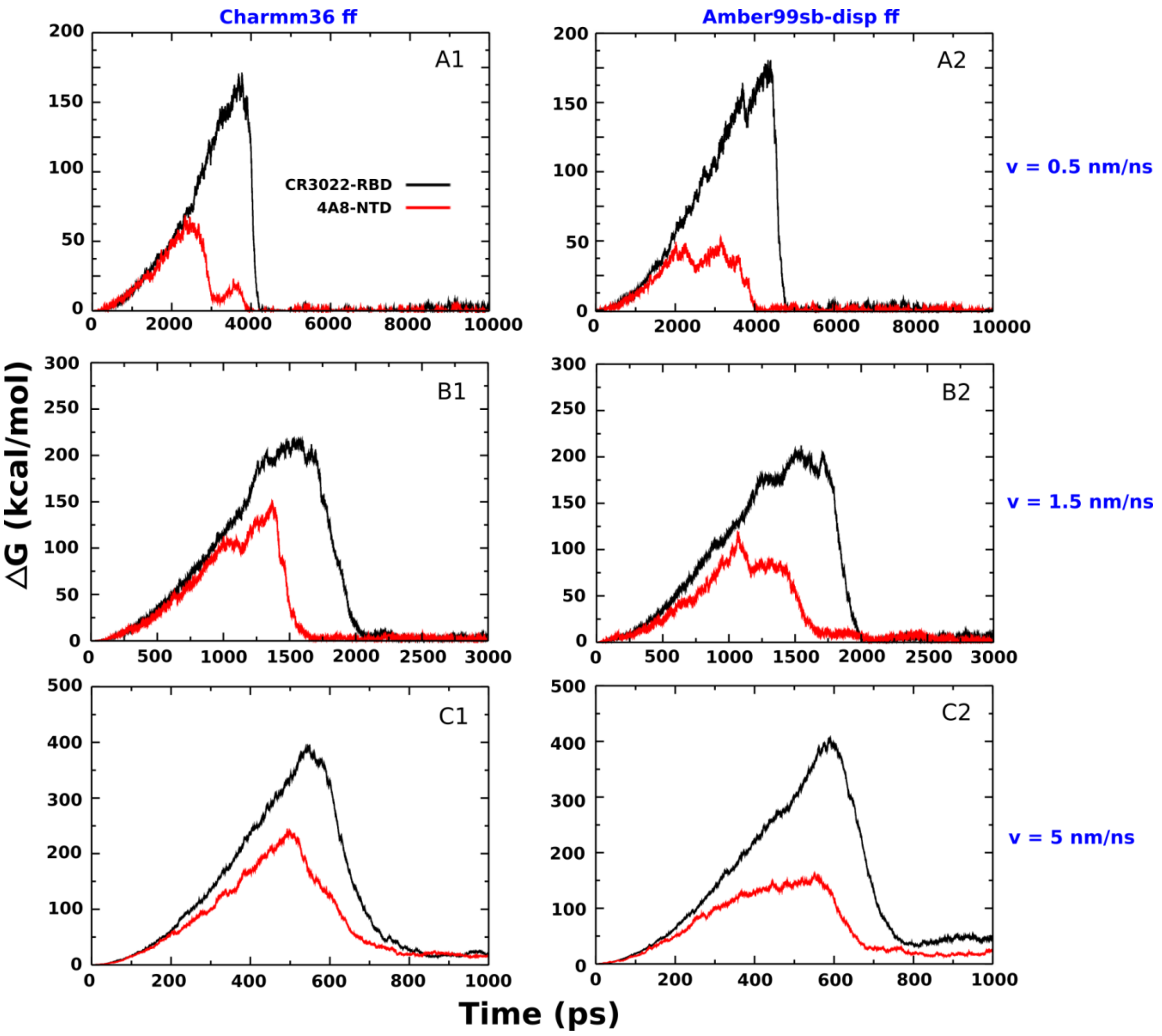

Figure S5. Time dependence of the non-equilibrium free energy of CR3022-RBD and 4A8-NTD, obtained at v = 0.5, v = 1.5 and v = 5 nm/ns using the CHARMM 36 (left) and AMBER99SB-DISP (right) force fields. The results were obtained from five independent SMD runs.

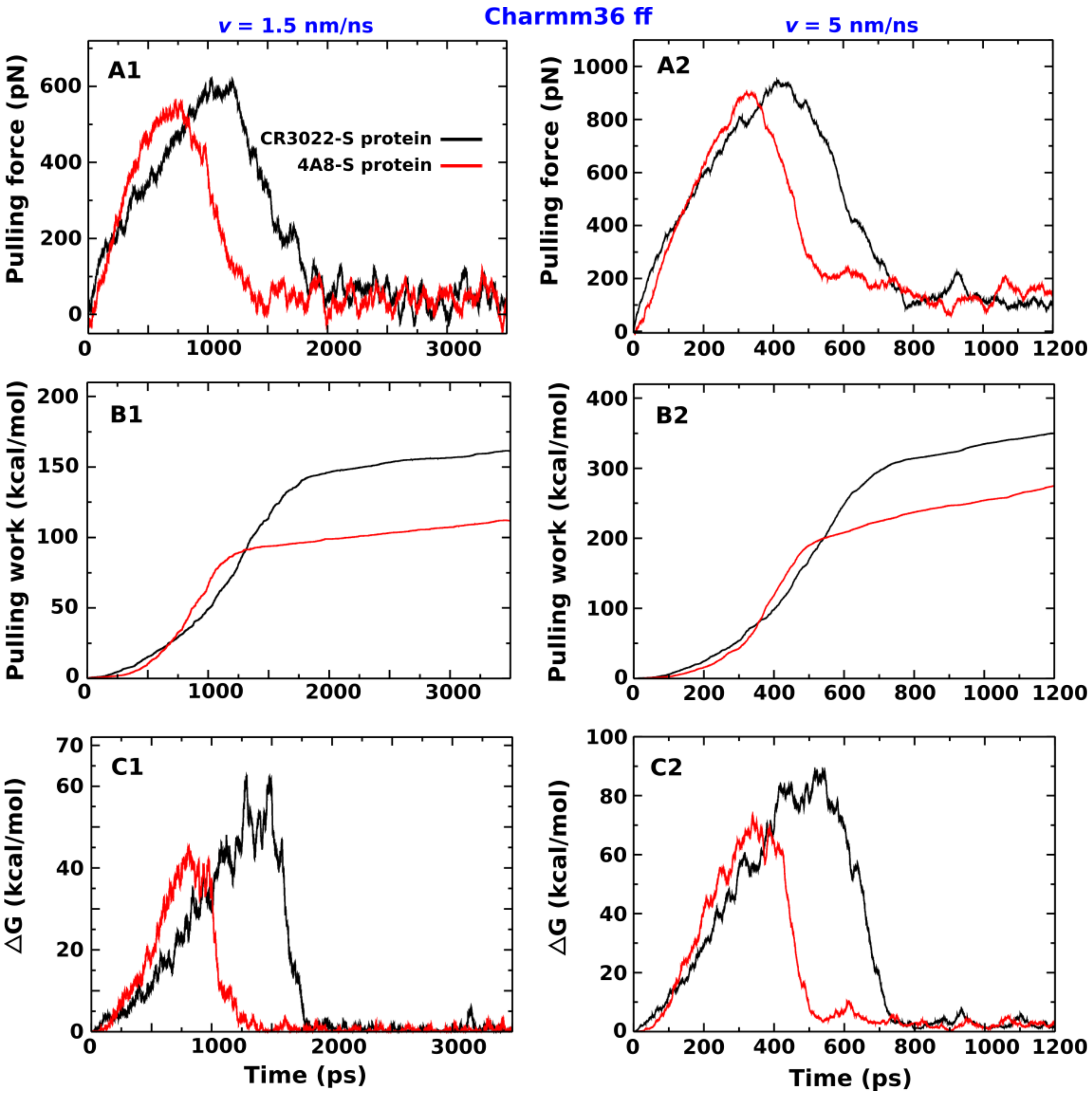

Figure S6. The time dependence of the pulling force, work and non-equilibrium free energy of CR3022-S protein and 4A8-S protein complexes. The results were averaged over five independent SMD runs at v = 1.5 and v = 5 nm/ns. The CHARMM 36 force field was used.

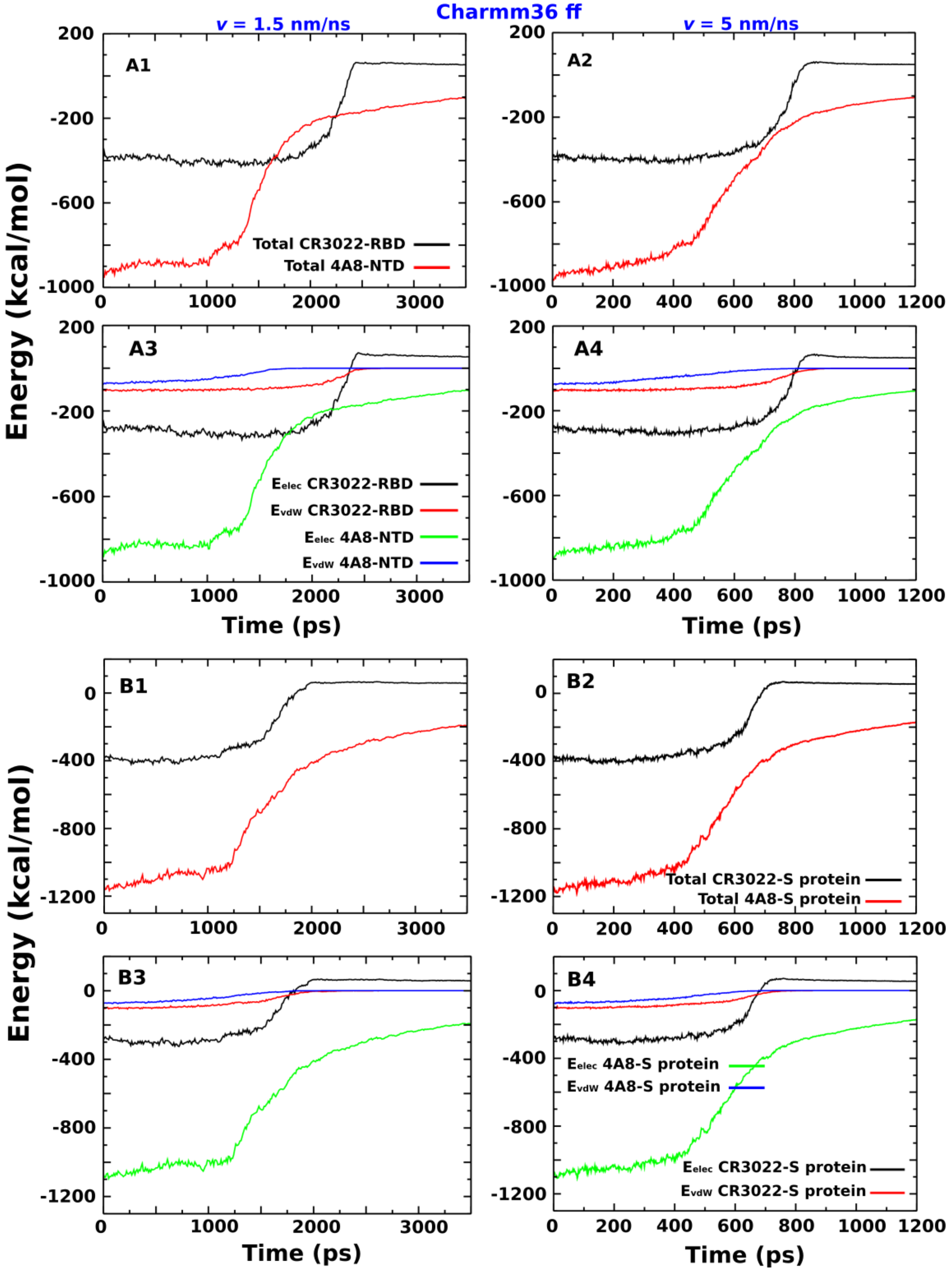

Figure S7. The time dependence of the total interaction energy (sum of electrostatic and vdW) (A1 and A2), electrostatic and vdW interaction energies (A3 and A4) for CR3022-RBD and 4A8-NTD complexes. B1, B2, B3 and B4 are the same as A1, A2, A3 and A4, but for CR3022-S protein and 4A8-S protein complexes. The results were obtained from five independent SMD runs at $v = 1.5$ and $v = 5$ nm/ns. The CHARMM 36 force field was used.

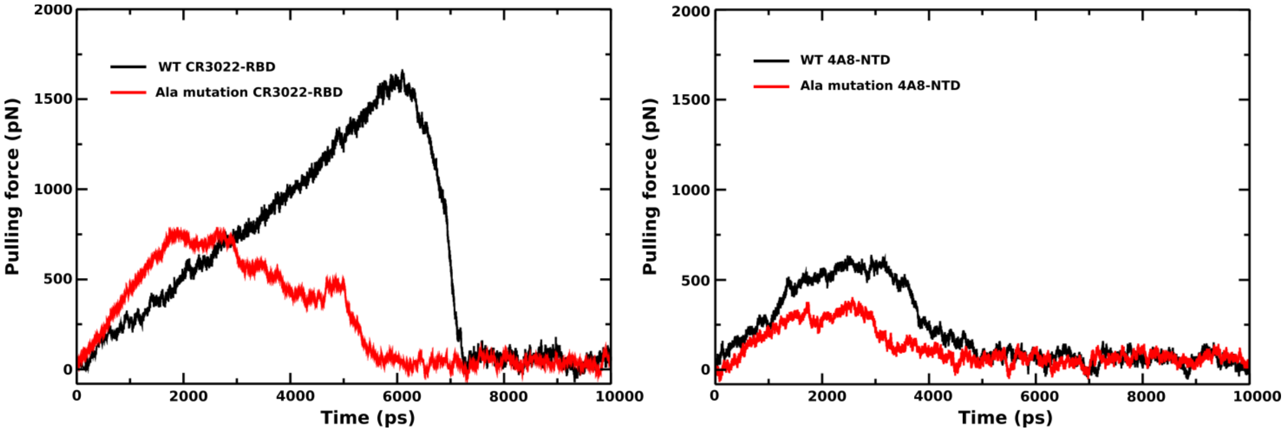

Figure S8. Time dependence of the pulling force for the CR3022-RBD and 4A8-NTD complexes (black) and these complexes, but the most important charged residues in the binding region have been replaced by Alanine (red). Results were obtained at v = 0.5 nm/ns using the CHARMM 36 force field.

Table S1: The rupture force (F_{max}), unbinding time (t_{max}), work of external force (W), non-equilibrium binding (ΔG_{bind}) and unbinding (ΔG_{unbind}) free energy barriers were obtained for both CR3022-RBD and 4A8-NTD complexes. The results were obtained from five independent SMD trajectories at pulling speed v = 0.5, 1.5 and 5 nm/ns with CHARMM 36 and AMBER99SB-DISP force files. The errors represent standard deviations.

**Charmm36 ff**										
**Pulling speed *v* *(nm/ns)***	**F_{max}** (pN)		**t_{max}** (ps)		**W** (kcal/mol)		**ΔG_{bind}** (kcal/mol)		**ΔG_{unbind}** (kcal/mol)	
	CR3022-RBD	4A8-NTD	CR3022-RBD	4A8-NTD	CR3022-RBD	4A8-NTD	CR3022-RBD	4A8-NTD	CR3022-RBD	4A8-NTD
**0.5**	1082.7 ± 125.1	834.1 ±110.3	2884.4 ±266.3	2459.7 ±77.5	199.7 ±50.3	143.1 ±16.0	168.3 ±27.8	68.9 ±13.3	171.3 ±30.3	69.0 ±13.3
**1.5**	1303.3 ±289.6	1018.4 ±169.2	1259.5 ±247.9	1046.4 ±149.2	361.0 ±82.5	242.6 ±34.2	216.3 ±34.9	149.1 ±25.8	218.9 ±36.8	150.7 ±26.7
**5**	1740.9 ±180.1	1392.0 ±154.9	450.5 ±47.5	427.7 ±57.5	631.9 ±92.2	539.5 ±60.1	370.9 ±34.0	223.9 ±31.7	391.4 ±36.5	240.1 ±33.9
**Amber99sb-disp ff**										
**Pulling speed *v* *(nm/ns)***	**F_{max}** (pN)		**t_{max}** (ps)		**W** (kcal/mol)		**ΔG_{bind}** (kcal/mol)		**ΔG_{unbind}** (kcal/mol)	
	CR3022-RBD	4A8-NTD	CR3022-RBD	4A8-NTD	CR3022-RBD	4A8-NTD	CR3022-RBD	4A8-NTD	CR3022-RBD	4A8-NTD
**0.5**	1345.8 ±91.9	684.0 ±63.7	3741.1 ±264.4	1983.1 ±419.7	283.4 ±40.6	144.4 ±57.6	181.1 ±18.1	52.9 ±8.3	180.7 ±18.0	53.3 ±10.2
**1.5**	1487.0 ±165.6	915.3 ±169.2	1277.0 ±183.3	1079.9 ±171.6	387.8 ±54.4	252.1 ±30.1	197.6 ±28.4	115.6 ±24.3	209.2 ±30.5	117.8 ±25.6
**5**	1855.2 ±88.2	1189.2 ±234.9	455.0 ±59.5	446.0 ±61.2	692.9 ±81.5	501.5 ±75.2	358.0 ±35.1	138.1 ±30.3	405.3 ±38.7	162.1 ±33.1

Table S2: The η values determined for stability of protein domains and interfaces, as well as for 4A8-NTD and CR3022-RBD interactions in CG simulations.

	**4A8**	**CR3022**	**SARS-CoV-2 NTD (NTD)**	**SARS-CoV-2 RBD (RBD)**	**4A8-NTD**	**CR3022-RBD**
**η**	Chain L: 2.480<br>Chain H: 2.480<br>Interface: 2.124	Chain L: 2.480<br>Chain H: 2.480<br>Interface: 2.124	1.442	1.359	1.4	1.4
